# Pseudorabies virus induces ferroptosis by disrupting iron homeostasis through activation of TfR1 and ferritinophagy

**DOI:** 10.1128/jvi.00974-25

**Published:** 2025-09-02

**Authors:** Zicheng Ma, Lei Guo, Ran Ji, Zhe Sun, Jingyi Wang, Yang Yan, Kesen Liu, Hanhua Zhang, Xingya Wang, Chengyue Wu, Lin Lian, Wandi Cao, Juan Bai, Ping Jiang, Xing Liu

**Affiliations:** 1 Key Laboratory of Animal Diseases Diagnostic and Immunology, Ministry of Agriculture, MOE International Joint Collaborative Research Laboratory for Animal Health & Food Safety, College of Veterinary Medicine, Nanjing Agricultural University 261674, Nanjing, China; 2 College of Life Sciences, Nanjing Agricultural University 98430, Nanjing, China; 3 Jiangsu Co-Innovation Center for Prevention and Control of Important Animal Infectious Diseases and Zoonoses, Yangzhou, China; Lerner Research Institute, Cleveland Clinic, Cleveland, Ohio, USA

**Keywords:** programmed cell death, ferroptosis, PRV, iron homeostasis, TfR1, ferritinophagy

## Abstract

Programmed cell death (PCD) refers to a regulated cellular process involving a cascade of biochemical reactions and molecular mechanisms, commonly including apoptosis, necroptosis, and pyroptosis. Ferroptosis is a recently identified form of PCD distinguished by its dependence on iron. Emerging evidence underscores the significance of ferroptosis in viral infections; however, its role in Pseudorabies virus (PRV) infection, an enveloped double-stranded DNA virus belonging to the Alphaherpesvirinae subfamily, remains poorly understood. Here, we demonstrate that PRV infection induces multiple forms of PCD, including ferroptosis, which is characterized by mitochondrial shrinkage, lipid peroxidation, ferrous iron (Fe²^+^) accumulation, and elevated levels of reactive oxygen species (ROS). Ferroptosis facilitates PRV replication, with iron overload playing a crucial role. Mechanistically, we show that transferrin receptor 1 (TfR1) and ferritinophagy are involved in PRV-induced iron overload. Specifically, PRV infection upregulates TfR1 expression via hypoxia-inducible factor-1β (HIF-1β) and promotes its translocation to the cell membrane through Rab11a, thereby enhancing the cellular import of extracellular ferric iron (Fe³^+^). In parallel, PRV activates ferritinophagy to degrade ferritin heavy chain 1 (FTH1) via selective autophagy receptors, nuclear receptor coactivator 4 (NCOA4) and Tax1-binding protein 1 (TAX1BP1), further contributing to intracellular iron accumulation. Altogether, these findings demonstrate that PRV induces ferroptosis by disrupting iron homeostasis through TfR1 activation and ferritinophagy induction, providing novel insights into the pathogenesis of PRV and other herpesviruses.

**IMPORTANCE:**

Ferroptosis is an iron-dependent form of non-apoptotic cell death that primarily involves iron overload, lipid peroxidation, and suppression of antioxidant systems. Increasing evidence indicates that ferroptosis plays an important role in viral infections. In this study, we show that PRV induces ferroptosis by disrupting iron homeostasis through TfR1 activation and ferritinophagy induction. On one hand, PRV infection upregulates TfR1 expression through HIF-1β and facilitates TfR1 translocation to the cell membrane via Rab11a, leading to enhanced import of extracellular Fe^3+^ into cells. On the other hand, PRV exploits the selective autophagy receptors NCOA4 and TAX1BP1, which strengthens the interaction between NCOA4, TAX1BP1, and FTH1, triggering ferritinophagy and increasing intracellular Fe^2+^ levels. Collectively, these findings enrich the understanding of the mechanism by which PRV induces ferroptosis, shedding new light on PRV and other alpha-herpesvirus infections.

## INTRODUCTION

Cell death is defined as the irreversible damage to cells caused by lethal disruptions in their metabolism, structure, or function (1). It is broadly classified into two types based on the underlying mechanisms: programmed cell death (PCD) and accidental cell death (ACD) (2). Compared to ACD, the process of PCD is tightly regulated and is governed by specific molecules and signaling pathways. As a key host defense mechanism, PCD plays an essential role in eliminating virus-infected cells and includes well-characterized forms such as apoptosis, necroptosis, and pyroptosis (3). Ferroptosis is a recently identified form of PCD that is driven by the iron-dependent accumulation of lipid-based ROS and lipid peroxides (4–7). Morphologically, ferroptosis is characterized by reduced mitochondrial volume, diminished or absent mitochondrial cristae, elevated intracellular Fe²^+^ levels, increased lipid peroxidation, and excessive ROS production (8, 9). The core molecular mechanism involves enhanced oxidative stress and the suppression of antioxidant defenses. Mechanistically, oxidative damage in ferroptosis can be attributed to two interconnected processes: lipid peroxidation and iron overload.

Iron overload results from imbalances in iron metabolism, including altered iron absorption, storage, and efflux (10). Extracellular Fe^3+^ is primarily imported into cells via the membrane protein TfR1 and subsequently trafficked into the endosome (11). Within endosomes, Fe^3+^ is reduced to Fe²^+^ by ferric reductases and released into the cytoplasmic labile iron pool (12). In the cytoplasm, excess iron is sequestered in ferritin, a protein complex composed of FTH1 and ferritin light chain (FTL). Ferritin can bind to NCOA4, a selective cargo receptor that mediates ferritin autophagy termed ferritinophagy, thereby releasing large amounts of Fe²^+^ into the cytoplasm (5, 13). The export of intracellular Fe²^+^ is mediated by the membrane transporter ferroportin (FPN). When intracellular Fe²^+^ levels rise, cells generate high levels of hydroxyl free radicals through the Fenton reaction, which promotes the peroxidation of polyunsaturated fatty acids (PUFAs) (14). Under normal conditions, cells are protected from ROS and lipid peroxides through antioxidant systems. However, when these systems are suppressed, cells become highly susceptible to ferroptosis (14).

In recent years, increasing evidence has highlighted a strong link between ferroptosis and various viral infections (15, 16). Ferroptosis can serve as a host defense mechanism against viruses or, conversely, be exploited by viruses to facilitate their replication and pathogenesis (17). For example, Epstein-Barr virus (EBV) reduces cellular sensitivity to ferroptosis by activating the sequestosome 1/kelch-like ECH-associated protein 1/nuclear factor erythroid 2-related factor 2 (p62/Keap1/Nrf2) signaling pathway and upregulating the expression of solute carrier family 7 member 11 (SLC7A11) and glutathione peroxidase 4 (GPX4) (18, 19). In contrast, swine influenza virus (SIV) downregulates SLC7A11, leading to suppressed GPX4 expression and activity, ultimately triggering ferroptosis (20). Hepatitis B virus (HBV) also contributes to ferroptosis: its oncoprotein HBx downregulates SLC7A11 through EZH2-mediated transcriptional repression, promoting ferroptosis in acute liver failure (21). Newcastle disease virus (NDV) induces ferroptosis by activating p53 to repress the expression of SLC7A11, leading to GPX4 suppression (22). Additionally, Severe Acute Respiratory Syndrome Coronavirus-2 (SARS-CoV-2) infection causes significant expression of ferroptosis-associated genes, inducing ferroptosis by downregulating GPX4 and decreasing glutathione (23, 24). In alpha-herpesvirus, Herpes simplex virus 1 (HSV-1) infection of astrocytes and microglia leads to increased intracellular Fe²^+^ concentrations and ROS accumulation, inducing ferroptosis and exacerbating the severity of viral encephalitis (25).

PRV, a member of the alpha-herpesvirus subfamily, poses a significant and devastating threat to the swine industry (26, 27). Moreover, similar to other alpha-herpesviruses such as varicella-zoster virus (VZV) and HSV-1/2 (28, 29), PRV can infect humans and induce acute encephalitis (30–32). A recent study has shown that HSV-1 disturbs cellular redox homeostasis through the Nrf2 pathway and promotes ferroptosis (25). In this study, we demonstrate that PRV induces ferroptosis by disrupting iron metabolism, thereby facilitating its replication. These findings uncover a novel mechanism linking PCD and alpha-herpesvirus infection and offer new insights into the pathogenesis of large DNA viruses.

## RESULTS

### PRV infection induces ferroptosis

To preliminarily investigate the relationship between PRV infection and PCD, we determined the key indicators of apoptosis, necroptosis, and pyroptosis. The results showed that increasing the PRV infection dose led to a gradual increase in apoptosis levels (Fig. S1A). Further analysis revealed a slight increase in p-MLKL and the presence of mildly cleaved Gasdermin D (GSDMD) at a high MOI (multiplicity of infection), although these changes were not significant, and no cleaved form of Gasdermin E (GSDME) was observed (Fig. S1B).

**Fig 1:**
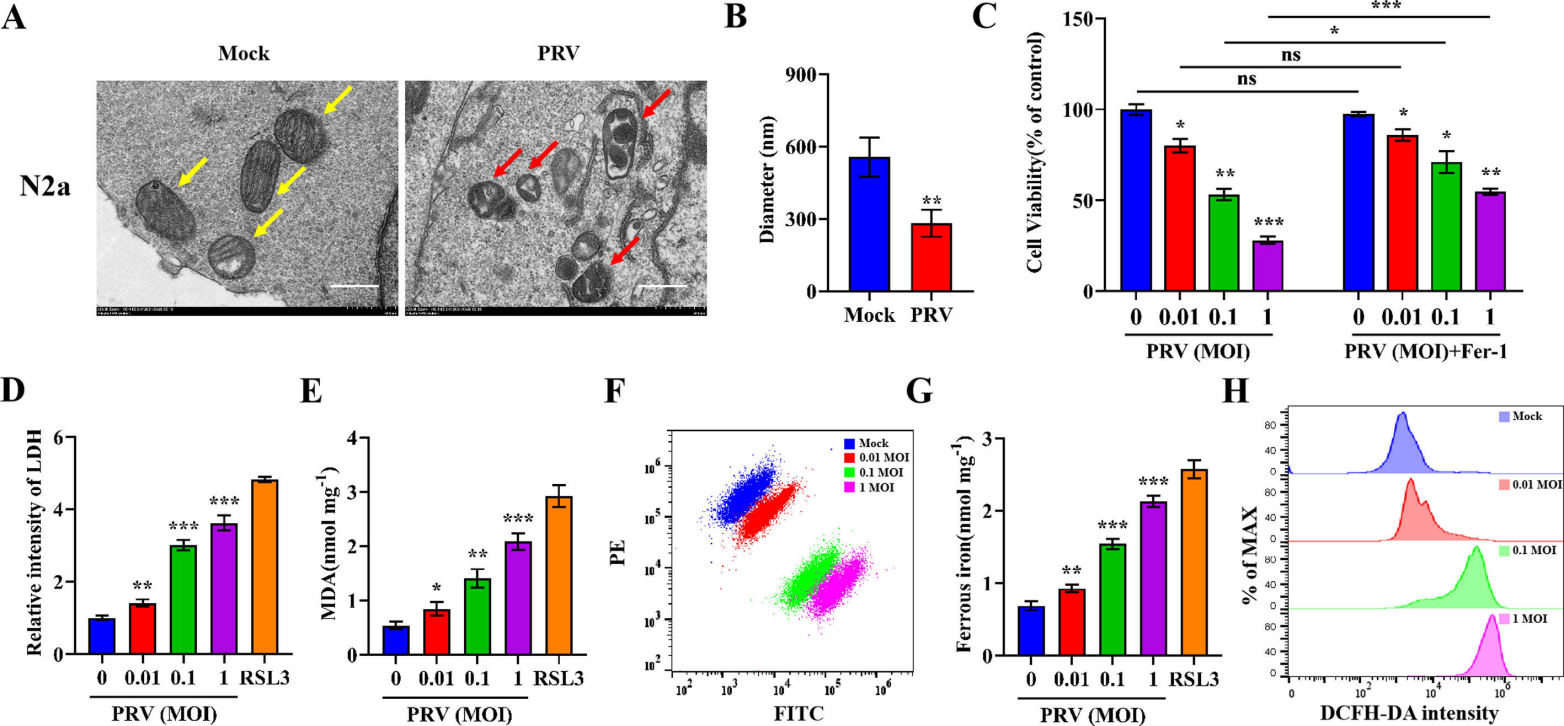
PRV induces ferroptosis. (**A**) N2a cells were mock- or PRV-infected (MOI = 0.1) for 24 h, and images were obtained by using a transmission electron microscope at 20,000 × magnification. Yellow arrows indicate normal mitochondria in mock-infected cells, and red arrows indicate mitochondrial atrophy in PRV-infected cells. Representative images are shown. Scale bar, 500 nm. (**B**) The diameters of mitochondria in N2a cells were quantified. (**C**) N2a cells were treated with Fer-1 or vehicle (DMSO) for 2 h, followed by PRV infection (MOI = 0.01, 0.1, or 1) or mock infection and additional treatment of Fer-1 (80 µM) or vehicle. At 24 hpi, cell viability was determined by CCK-8 assay, with the level of cell viability in cells treated with vehicle defined as 100%. N2a cells were mock- or PRV-infected (MOI = 0.01, 0.1, or 1) for 24 h or treated with RSL3 (10 µM) as positive control. (**D**) LDH released into the supernatants was determined by a cytotoxicity assay, with LDH in mock-infected cells defined as 1. (**E**) MDA concentrations in cell lysates were determined using MDA assay. (**F**) Lipid peroxidation levels in cells were measured using the fluorescent probe C11-BODIPY 581/591. (**G**) Ferrous iron concentrations in cell lysates were determined using ferrous iron colorimetric assay. (**H**) ROS levels in cells were determined using a ROS assay. The data shown are means ± SD. *, *P* < 0.05; **, *P* < 0.01; ***, *P* < 0.001. The experimental data are representative of results from three independent experiments.

Ferroptosis, a recently identified iron-dependent form of PCD, plays a critical role in viral replication and pathogenesis (17). To determine whether PRV infection induces ferroptosis, we analyzed mitochondrial morphology in mock-infected and PRV-infected N2a cells using transmission electron microscopy. PRV-infected cells exhibited swollen mitochondria with reduced cristae compared to uninfected cells (Fig. 1A) and showed significantly smaller mitochondrial diameters (Fig. 1B). Next, we treated cells with increasing doses of PRV and observed a corresponding decrease in cell viability, which was rescued by Ferrostatin-1 (Fer-1), a specific ferroptosis inhibitor (33, 34) (Fig. 1C). As a positive control, we used RSL3, a known ferroptosis inducer, and found that PRV infection significantly increased levels of lactate dehydrogenase (LDH) release, malondialdehyde (MDA), Fe^2+^, and ROS (Fig. 1D, E, G and H). To assess lipid peroxidation, we used the fluorescent probe C11-BODIPY 581/591. In its reduced form, C11-BODIPY emits red fluorescence, which shifts to green upon oxidation, enabling real-time detection of lipid peroxidation. Our results showed that PRV infection induced dose-dependent lipid peroxidation (Fig. 1F). Similar results were observed in two additional cell lines: PK-15 (Fig. S2A through H) and SH-SY5Y cells (Fig. S2I through P). In contrast, PRV inactivated by ultraviolet irradiation (PRV-UV) failed to induce ferroptosis (Fig. S3A through G). Together, these findings demonstrate that PRV infection can induce ferroptosis.

### Ferroptosis promotes PRV replication

**Fig 2:**
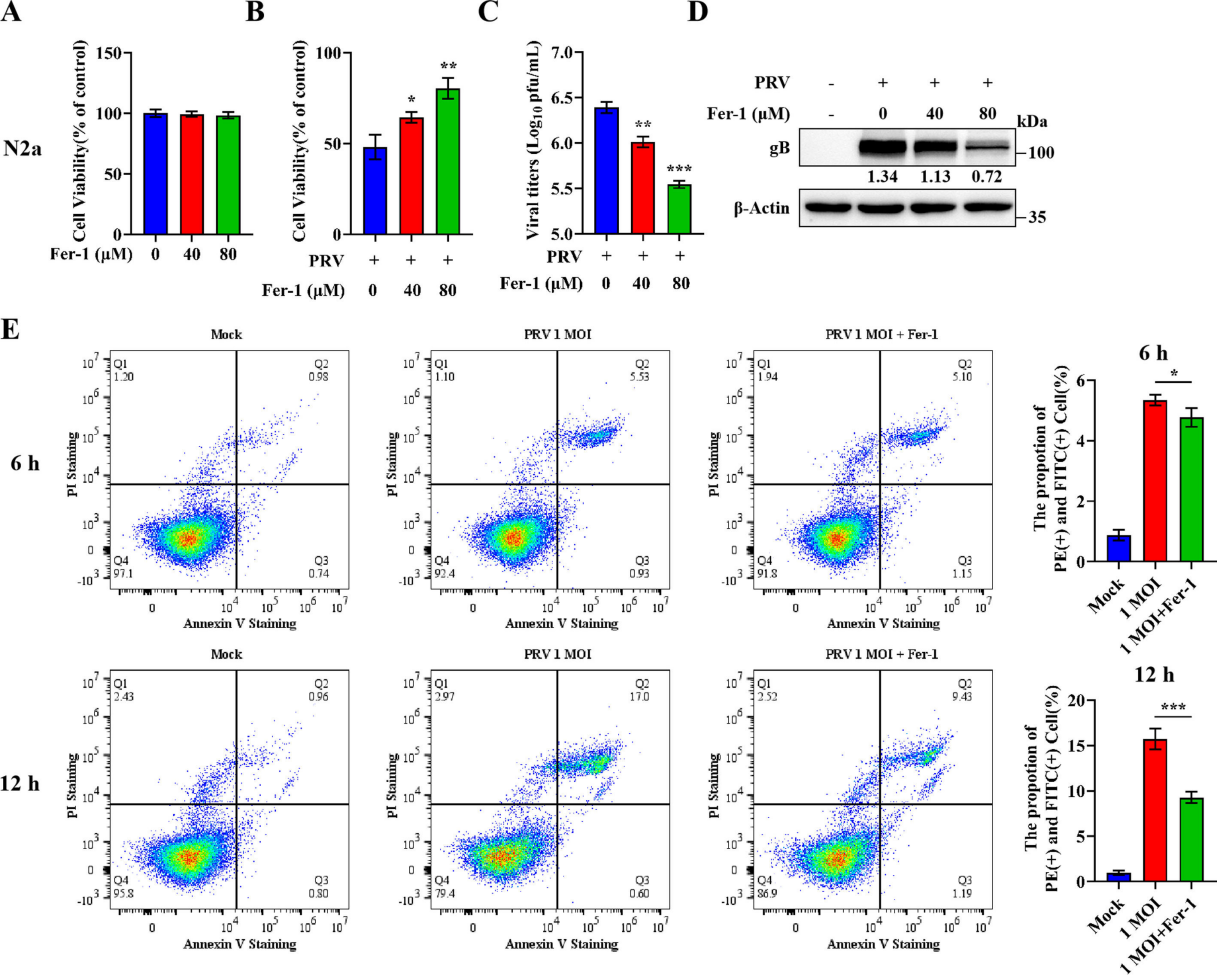
Ferroptosis promotes PRV replication. (**A**) Cells were treated with different concentrations of Fer-1 (40 or 80 µM) or vehicle (DMSO) for 24 h. Cell viability of N2a cells was determined by CCK-8 assay, with the level of cell viability in cells treated with vehicle defined as 100%. (**B**) N2a cells were treated with Fer-1 or vehicle (DMSO) for 2 h, followed by PRV infection (MOI = 0.1) or mock infection and additional treatment of Fer-1 (80 µM) or vehicle. At 24 hpi, cell viability was determined by CCK-8 assay, with the level of cell viability in cells treated with vehicle defined as 100%. (**C**) At 24 hpi, cells and supernatants were harvested, and virus titers were determined in Vero cells using a plaque assay. (**D**) Cell lysates were analyzed by western blot for gB and β-actin. The protein levels were quantified by Image J and normalized to β-actin. (**E**) N2a cells were treated with Fer-1 or vehicle (DMSO) for 2 h, followed by PRV infection or mock infection and additional treatment of Fer-1 or vehicle. At 6 or 12 hpi, apoptosis was assessed using Annexin V-FITC/PI staining and analyzed by fluorescence-activated cell sorting (FACS). The values in each panel represent the percentage of viable cells. The data shown are means ± SD. *, *P* < 0.05; **, *P* < 0.01; ***, *P* < 0.001. The experimental data are representative of results from three independent experiments.

Given the emerging nature of ferroptosis, its role in PRV infection was subsequently determined. To investigate the role of ferroptosis in PRV replication, N2a cells were treated with varying concentrations of Ferrostatin-1 (Fer-1), a specific ferroptosis inhibitor, followed by PRV infection. Importantly, Fer-1 treatment at the tested doses did not affect cell viability under normal conditions (Fig. 2A). However, under PRV infection, Fer-1 significantly increased cell viability in a dose-dependent manner compared to the vehicle control (DMSO) (Fig. 2B). Correspondingly, viral titers were reduced in a dose-dependent manner following Fer-1 treatment (Fig. 2C), and expression of the viral protein gB gradually declined with increasing Fer-1 concentrations (Fig. 2D). Similar results were observed in PK-15 (Fig. S4A through D) and SH-SY5Y cells (Fig. S4E through H). We also examined the effect of Fer-1 on apoptosis. Flow cytometry analysis revealed that although apoptosis levels increased over time in both treatment groups during PRV infection, cells treated with Fer-1 exhibited a significantly greater reduction in apoptosis compared to the DMSO control group, particularly at later time points (Fig. 2E). These findings suggest that the ferroptosis inhibitor Fer-1 can suppress both PRV-induced cell death and viral replication, indicating that PRV-induced ferroptosis contributes to efficient viral replication.

### PRV replication depends on extracellular iron concentration

**Fig 3:**
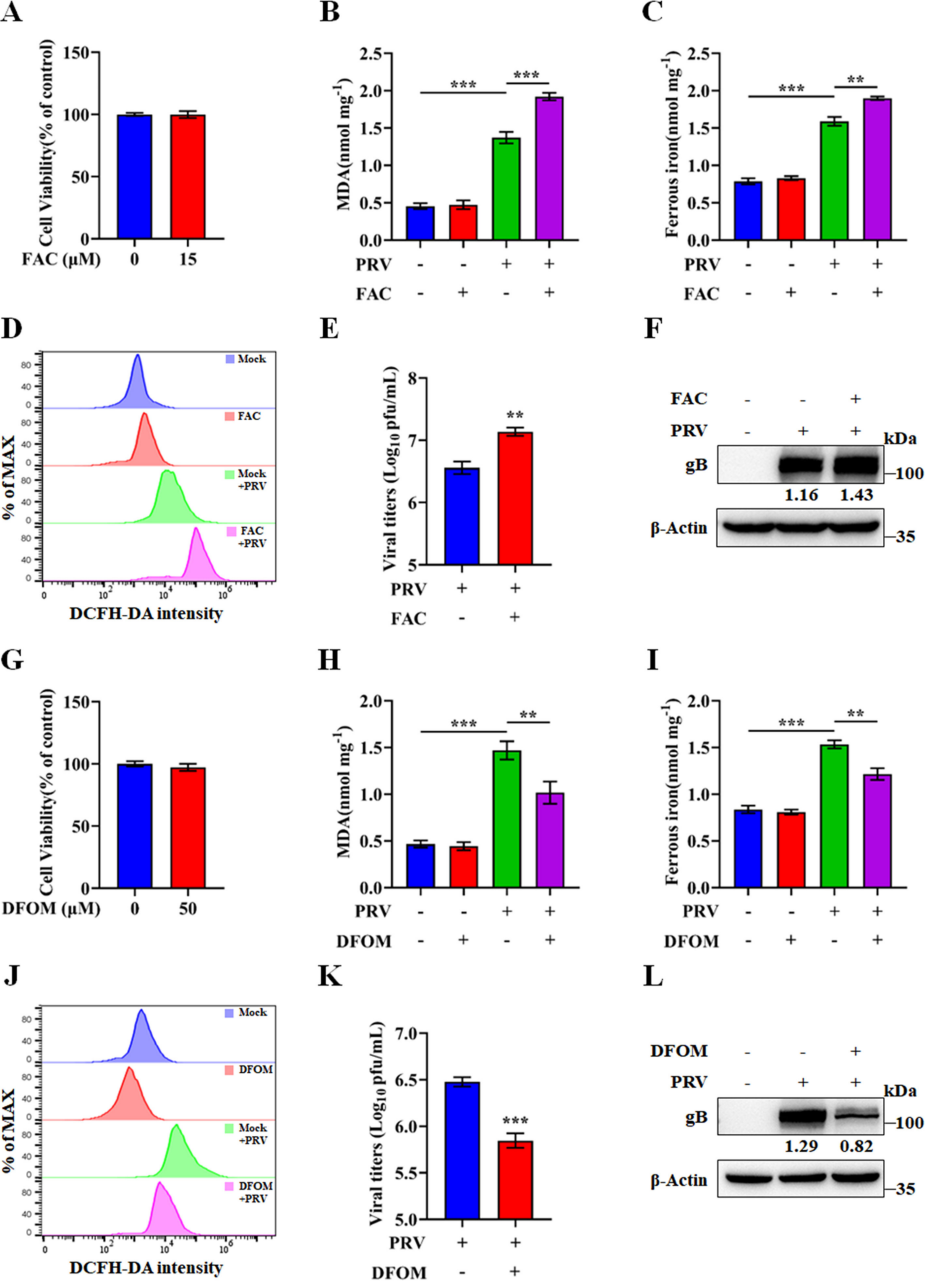
PRV replication depends on extracellular iron concentration. N2a cells were treated with either 15 µM FAC, 50 µM DFOM, or vehicle (DMSO) for 2 h, followed by PRV infection (MOI = 0.1) or mock infection and additional treatment of FAC/DFOM or vehicle. At 24 hpi, the following assays were performed: (**A, G**) Cell viability was determined using a CCK-8 assay, with vehicle-treated cells defined as 100%. (**B, H**) MDA concentrations in cell lysates were determined using MDA assay. (**C, I**) Ferrous iron concentrations in cell lysates were determined using a ferrous iron colorimetric assay. (**D, J**) ROS levels in cells were determined using a ROS assay. (**E, K**) Cells and supernatants were harvested, and virus titers were determined in Vero cells using a plaque assay. (**F, L**) Cell lysates were analyzed by Western blot for gB and β-actin. The protein levels were quantified using ImageJ and normalized to β-actin. The data shown are means ± SD. *, *P* < 0.05; **, *P* < 0.01; ***, *P* < 0.001. The experimental data are representative of results from three independent experiments.

We observed that the concentration of Fe^2+^ increased in a dose-dependent manner following PRV infection (Fig. 1G). To determine whether iron concentration influences PRV-induced ferroptosis and viral replication, we utilized ferric ammonium citrate (FAC), a non-transferrin-bound iron compound widely used to elevate extracellular iron levels (35, 36). N2a cells were treated with either FAC or vehicle (DMSO) and subsequently infected with PRV. FAC treatment had no significant impact on cell viability (Fig. 3A). To further elucidate the role of ferroptosis in PRV infection, we assessed key ferroptosis markers, including lipid peroxidation, Fe^2+^ levels, and ROS. In mock-infected N2a cells, FAC treatment did not significantly alter any of these markers compared to the DMSO control group. However, in PRV-infected cells, treated with FAC, lipid peroxidation, Fe²^+^, and ROS were significantly elevated relative to the control (DMSO) (Fig. 3B through D). Moreover, FAC treatment significantly increased both viral titers and expression of the viral envelope protein gB (Fig. 3E and F). To complement these findings, we performed parallel experiments using deferoxamine mesylate (DFOM), an iron chelator that reduces extracellular iron levels by forming stable iron complexes (37, 38). DFOM treatment did not affect cell viability at the concentrations used (Fig. 3G). However, it significantly reduced lipid peroxidation, Fe²^+^ levels, and ROS production, as well as viral titers and gB expression (Fig. 3H through L). Collectively, these results indicate that PRV-induced ferroptosis and viral replication are positively correlated with extracellular iron concentration.

### PRV infection induces ferroptosis by disrupting iron metabolism

**Fig 4:**
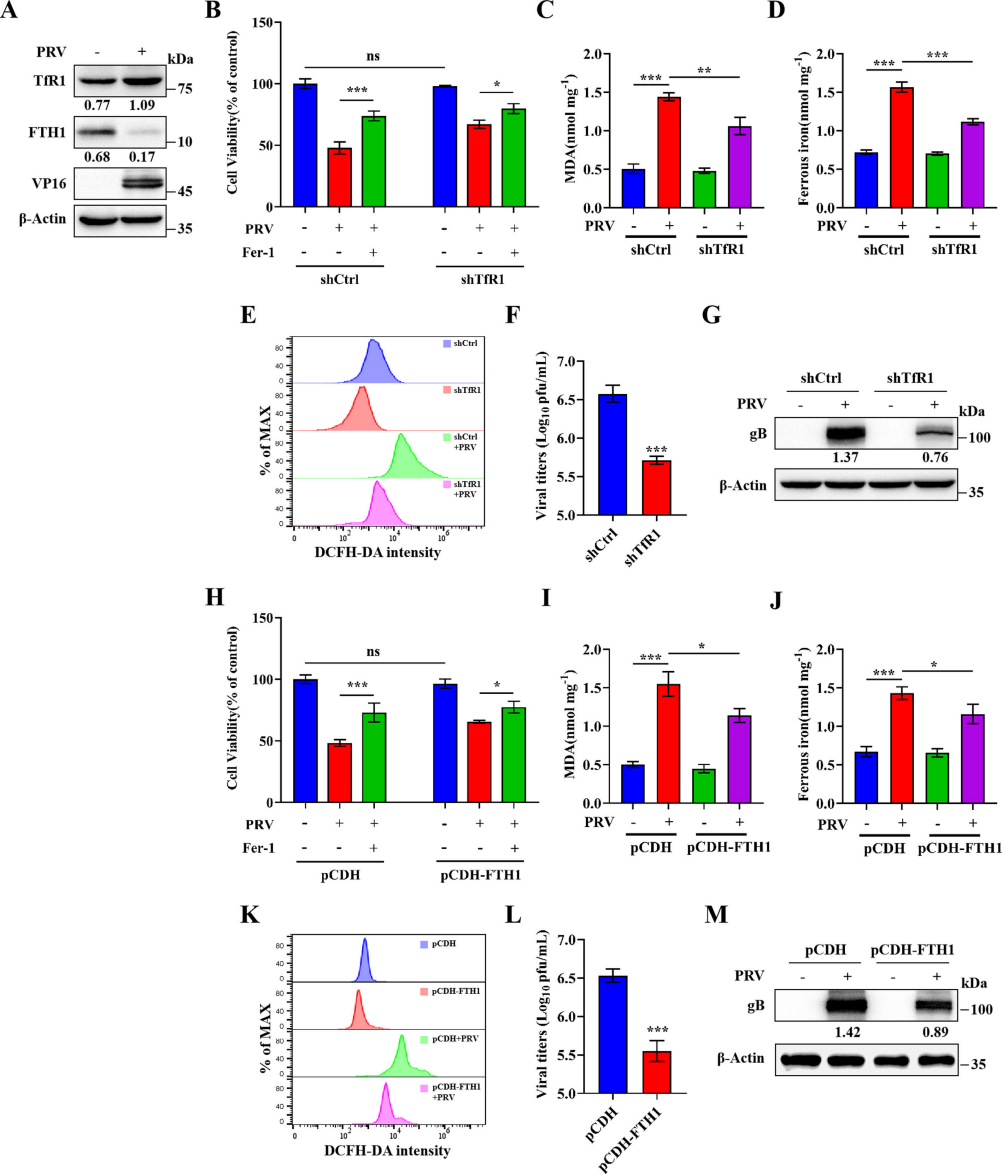
PRV infection induces ferroptosis through disruption of iron metabolism. (**A**) N2a cells were mock- or PRV-infected (MOI = 1) for 12 h. Cell lysates were analyzed by western blot for TfR1, FTH1, VP16, and β-actin. TfR1-knockdown (**B**), FTH1-overexpression (**H**), and wild-type N2a cells were treated with Fer-1 (80 µM) or the vehicle (DMSO) for 2 h, followed by PRV infection (MOI = 0.1) or mock infection and additional treatment of Fer-1 or vehicle. At 24 hpi, cell viability was determined by CCK-8 assay, with the level of cell viability in cells treated with vehicle defined as 100%. TfR1-knockdown, FTH1-overexpression and wild-type N2a cells were mock- or PRV-infected (MOI = 0.1) for 24 h. MDA concentrations in TfR1-knockdown (**C**) and FTH1-overexpression (**I**) cell lysates were determined. Ferrous iron concentrations in TfR1-knockdown (**D**) and FTH1-overexpression (**J**) cell lysates were determined. ROS levels in TfR1-knockdown (**E**) and FTH1-overexpression (**K**) cells were determined. Cells and supernatants were harvested, and total virus titers in TfR1-knockdown (**F**) and FTH1-overexpression (**L**) cells were determined in Vero cells using a plaque assay. Cell lysates of TfR1-knockdown (**G**) and FTH1-overexpression (**M**) were analyzed by western blot for gB and β-actin. The protein levels were quantified by Image J and normalized to β-actin. The data shown are means ± SD. *, *P* < 0.05; **, *P* < 0.01; ***, *P* < 0.001. The experimental data are representative of results from three independent experiments.

Since iron availability is critical for PRV-induced ferroptosis, we next investigated the relationship between PRV infection and iron metabolism. TfR1 is one of the major proteins mediating iron entry into cells and plays a key role in regulating cellular iron metabolism and maintaining iron balance (39). FTH1, a major subunit of ferritin, the primary intracellular iron storage complex, stores approximately 75% of newly imported iron and protects cells from iron overload and ferroptosis (40, 41). Degradation of FTH1 leads to the release of Fe²^+^, contributing to oxidative stress and ferroptosis (42). To determine whether PRV affects these pathways, we measured TfR1 and FTH1 expression following PRV infection. We observed that PRV infection significantly upregulated TfR1 expression while downregulating FTH1 expression (Fig. 4A). These alterations are consistent with the induction of ferroptosis and suggest a disruption of iron homeostasis during PRV infection.

To further explore the roles of TfR1 and FTH1 in PRV-induced ferroptosis, we knocked down TfR1 using short hairpin RNA (shRNA) and overexpressed FTH1 via lentiviral transduction in N2a cells. Knockdown and overexpression efficiencies were confirmed by RT-qPCR and western blot analysis (Fig. S5).

We then assessed cell viability, lipid peroxidation, Fe²^+^ levels, and ROS in PRV-infected wild-type and TfR1-knockdown cells. Ferrostatin-1 (Fer-1) effectively inhibited PRV-induced ferroptosis, and TfR1 knockdown significantly reduced ferroptosis-mediated cell death in infected cells (Fig. 4B). Correspondingly, TfR1-deficient cells exhibited reduced lipid peroxidation, Fe²^+^ levels, and ROS generation compared to wild-type controls (Fig. 4C through E). Importantly, the replication of PRV was significantly reduced, as evidenced by reduced viral titers and expression of viral protein gB (Fig. 4F and G). Parallel experiments in FTH1-overexpressing N2a cells showed similar protective effects. Upon PRV infection, FTH1-overexpressing cells exhibited decreased cell death, lipid peroxidation, Fe²^+^ accumulation, ROS production, and viral replication compared to wild-type cells (Fig. 4H through M).

Collectively, these findings demonstrate that PRV infection disrupts iron homeostasis through two complementary mechanisms: it upregulates TfR1 to enhance iron influx and downregulates FTH1 to promote intracellular Fe²^+^ release. These changes synergistically increase the labile iron pool, thereby facilitating ferroptosis and viral replication.

### HIF-1β promotes TfR1 mRNA expression following PRV infection

**Fig 5:**
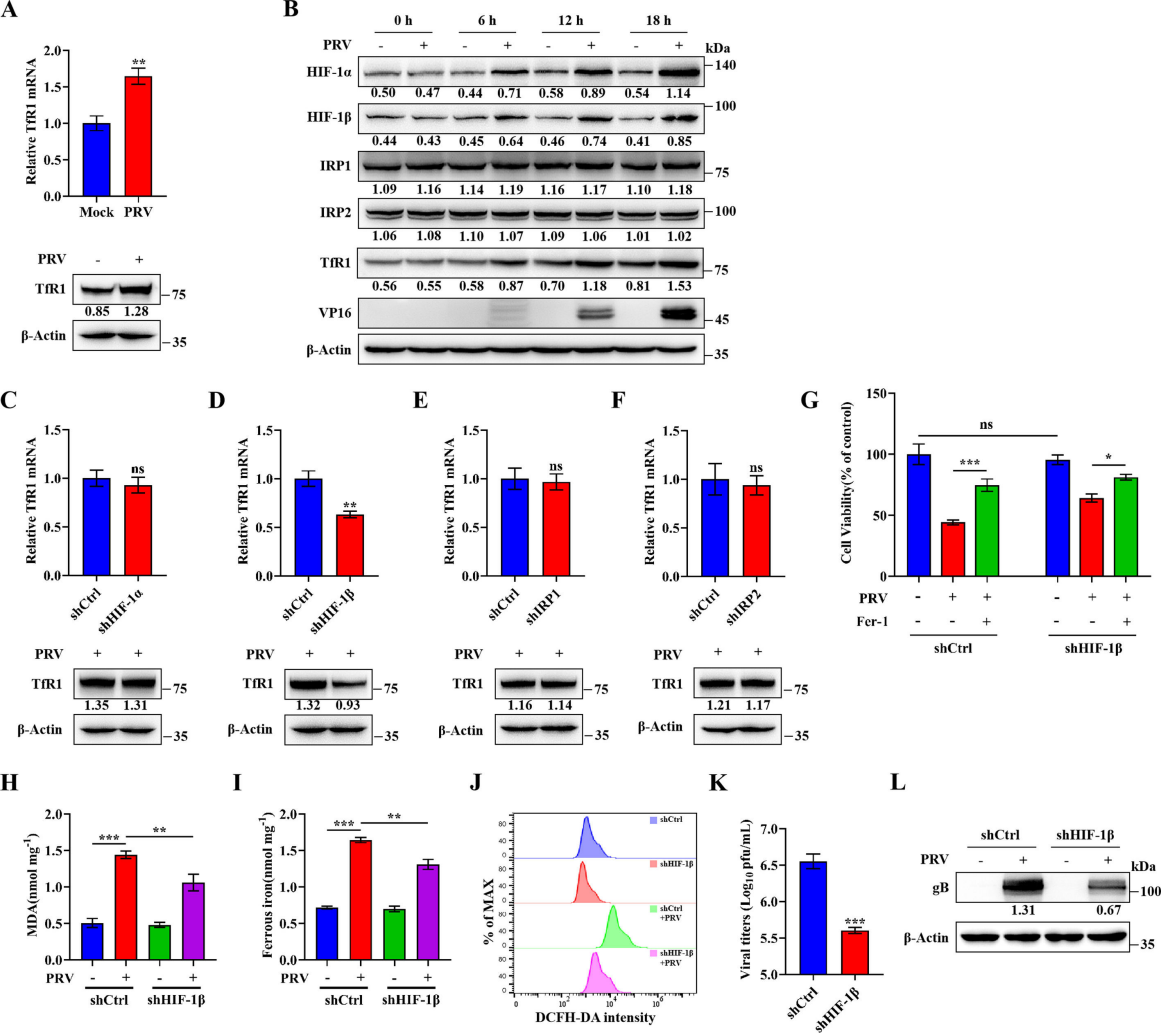
HIF-1β promotes TfR1 mRNA expression upon PRV infection. (**A**) N2a cells were mock- or PRV-infected (MOI = 1) for 12 h. RNA was extracted for RT-PCR analysis of TfR1 and RNA18s rRNA, and cell lysates were analyzed by Western blot for TfR1 and β-actin. (**B**) N2a cells were mock- or PRV-infected (MOI = 1) at various times. Cell lysates were analyzed by western blot for HIF-1α, HIF-1β, IRP1, IRP2, TfR1, VP16, and β-actin. HIF-1α-knockdown (**C**), HIF-1β-knockdown (**D**), IRP1-knockdown (**E**), IRP2-knockdown (**F**), and Wild-type N2a cells were harvested for RNA extraction and RT-PCR analysis of TfR1 and RNA18s rRNA, and cell lysates were analyzed by western blot for TFR1 and β-actin. (**G**) HIF-1β-knockdown cells and wild-type N2a cells were treated with Fer-1 or vehicle (DMSO) for 2 h, followed by PRV infection (MOI = 0.1) or mock infection and additional treatment of Fer-1 (80 µM) or vehicle. At 24 hpi, cell viability was determined by CCK-8 assay, with the level of cell viability in cells treated with vehicle defined as 100%. Wild-type and HIF-1β-knockdown N2a cells were mock- or PRV-infected (MOI = 0.1) for 24 h. (**H**) MDA concentrations in cell lysates were determined using MDA assay. (**I**) Ferrous iron concentrations in cell lysates were determined using a ferrous iron colorimetric assay. (**J**) ROS levels were determined using a ROS assay. (**K**) Cells and supernatants were harvested, and total virus titers were determined in Vero cells using a plaque assay. (**L**) Cell lysates were analyzed by western blot for gB and β-actin. The protein levels were quantified by Image J and normalized to β-actin. The data shown are means ± SD. *, *P* < 0.05; **, *P* < 0.01; ***, *P* < 0.001. The experimental data are representative of results from three independent experiments.

Next, we investigated the mechanism by which PRV infection upregulates TfR1 expression. TfR1 is regulated at the transcriptional, post-transcriptional, and post-translational levels (43). To determine whether PRV affects TfR1 transcription, we performed RT-qPCR and western blot analysis. We found that both TfR1 mRNA and protein levels were significantly increased upon PRV infection (Fig. 5A). Transcription of TfR1 is regulated by hypoxia-inducible factors (HIFs), which bind to hypoxia response elements (HREs) in the TfR1 promoter (43, 44). Post-transcriptionally, iron regulatory proteins (IRP1 and IRP2) stabilize TfR1 mRNA by binding to iron-responsive elements (IREs) in the 3′ untranslated region (3′-UTR), thereby preventing Regnase-1 (Reg-1)-mediated mRNA degradation (45). To assess the potential involvement of these pathways, we examined the expression of HIF-1α, HIF-1β, IRP1, and IRP2 at various time points following PRV infection. The levels of HIF-1α and HIF-1β increased over time, whereas IRP1 and IRP2 expression remained unchanged (Fig. 5B). To clarify their roles in PRV-mediated regulation of TfR1, we knocked down HIF-1α, HIF-1β, IRP1, and IRP2 in N2a cells. Knockdown efficiency was confirmed by RT-qPCR and western blot (Fig. S6). We then compared TfR1 mRNA and protein expression between knockdown and wild-type cells following PRV infection. Notably, HIF-1β knockdown led to a marked reduction in TfR1 mRNA and protein levels, while knockdown of HIF-1α, IRP1, or IRP2 had no significant effect (Fig. 5C through F). These results suggest that PRV induces TfR1 transcription through a HIF-1-dependent mechanism, with the HIF-1β subunit playing a critical role.

To further validate this hypothesis, we assessed cell viability, ferroptosis markers, and viral replication in HIF-1β-knockdown and wild-type N2a cells following PRV infection. HIF-1β knockdown significantly reduced cell death, lipid peroxidation, Fe²^+^ accumulation, ROS production (Fig. 5G through J), and viral replication (Fig. 5K and L). Collectively, these findings indicate that PRV promotes TfR1 expression via HIF-1β, leading to enhanced iron uptake and increased susceptibility to ferroptosis.

### PRV infection promotes endosomal trafficking of TfR1 via Rab11a

**Fig 6:**
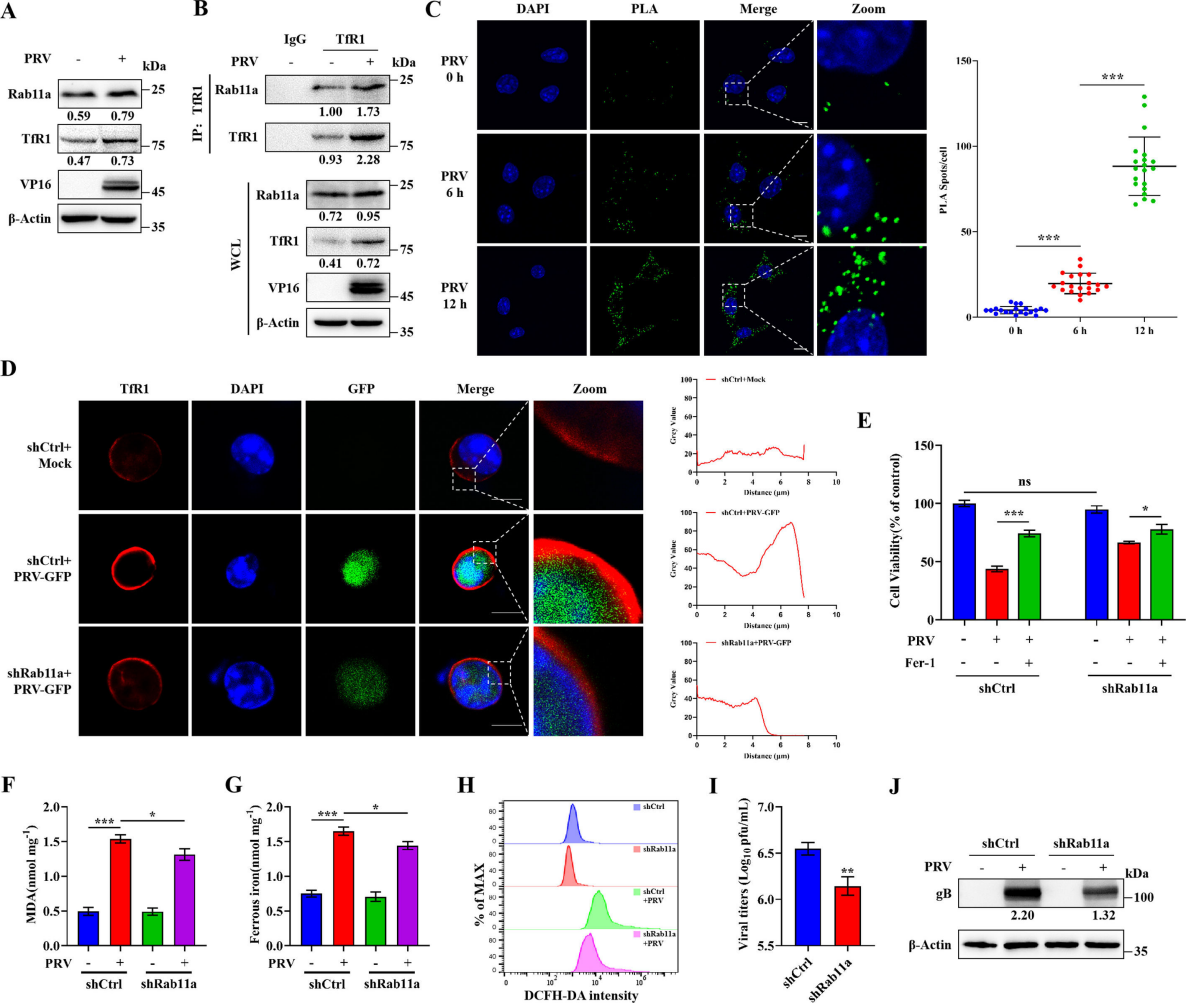
Rab11a promotes endosomal trafficking of TfR1 upon PRV infection. N2a cells were mock- or PRV-infected (MOI = 1) for 12 h. (**A**) Cell lysates were analyzed by western blot for Rab11a, TfR1, VP16, and β-actin. (**B**) Cell lysates were co-immunoprecipitated (co-IP) with anti-TfR1 antibody and then subjected to western blot analysis for Rab11a, TfR1, VP16, and β-actin. The grayscale intensity in the mock-infected group was set at 1.00 in the co-IP analysis. (**C**) N2a cells were mock-infected and infected with PRV at an MOI = 1 for 6 h or 12 h. Cells were stained for TfR1, Rab11a, and DAPI and performed using the Duolink *In Situ* Detection Reagents Green. Then observed by confocal microscopy. Scale bar, 10 µm. (**D**) Rab11a-knockdown and wild-type N2a cells were mock- or PRV-GFP-infected (MOI = 1) for 12 h. Cells were stained for TfR1and DAPI and observed by confocal microscopy. Scale bar, 10 µm. (**E**) Rab11a-knockdown cells and wild-type N2a cells were treated with Fer-1 (80 µM) or vehicle (DMSO) for 2 h, followed by PRV infection (MOI = 0.1) or mock infection and additional treatment of Fer-1 or vehicle. At 24 hpi, cell viability was determined by CCK-8 assay, with the level of cell viability in cells treated with vehicle defined as 100%. Wild-type and Rab11a-knockdown N2a cells were mock- or PRV-infected (MOI = 0.1) for 24 h. (**F**) MDA concentrations in cell lysates were determined using MDA assay. (**G**) Ferrous iron concentrations in cell lysates were determined using a ferrous iron colorimetric assay. (**H**) ROS levels were determined using a ROS assay. (**I**) Cells and supernatants were harvested, and total virus titers were determined in Vero cells using a plaque assay. (**J**) Cell lysates were analyzed by western blot for gB and β-actin. The protein levels were quantified by Image J and normalized to β-actin. The data shown are means ± SD. *, *P* < 0.05; **, *P* < 0.01; ***, *P* < 0.001. The experimental data are representative of results from three independent experiments.

TfR1 is a membrane protein that requires transport to the cell surface to perform its biological function, a process mediated by Rab11a (46, 47). To examine whether PRV affects Rab11a expression, we performed western blot analysis before and after PRV infection. The results showed a significant increase in Rab11a expression following infection (Fig. 6A). Moreover, co-immunoprecipitation and proximity ligation assay (PLA) experiments revealed that the interaction between TfR1 and Rab11a was enhanced upon PRV infection (Fig. 6B and C), likely due to the upregulation of both TfR1 and Rab11a expression in response to the virus.

To further assess the role of Rab11a in TfR1 trafficking during PRV infection, we knocked down Rab11a in N2a cells. Knockdown efficiency was confirmed by RT-qPCR and western blot analysis (Fig. S7A). Confocal microscopy revealed increased localization of TfR1 at the cell membrane following PRV-GFP infection, which was markedly reduced in Rab11a-knockdown cells (Fig. 6D). To test the functional consequences of Rab11a depletion, Rab11a-knockdown and wild-type N2a cells were infected with PRV, and subsequent ferroptosis and viral replication were evaluated. Rab11a knockdown led to reduced levels of cell death, lipid peroxidation, Fe^2+^ accumulation, and ROS production, as well as diminished PRV replication (Fig. 6E through J). Together, these findings suggest that PRV infection promotes Rab11a-dependent translocation of TfR1 to the cell membrane, enabling TfR1 to perform its function and thereby increasing cellular susceptibility to ferroptosis.

### PRV induces NCOA4 and TAX1BP1-mediated ferritinophagy

**Fig 7:**
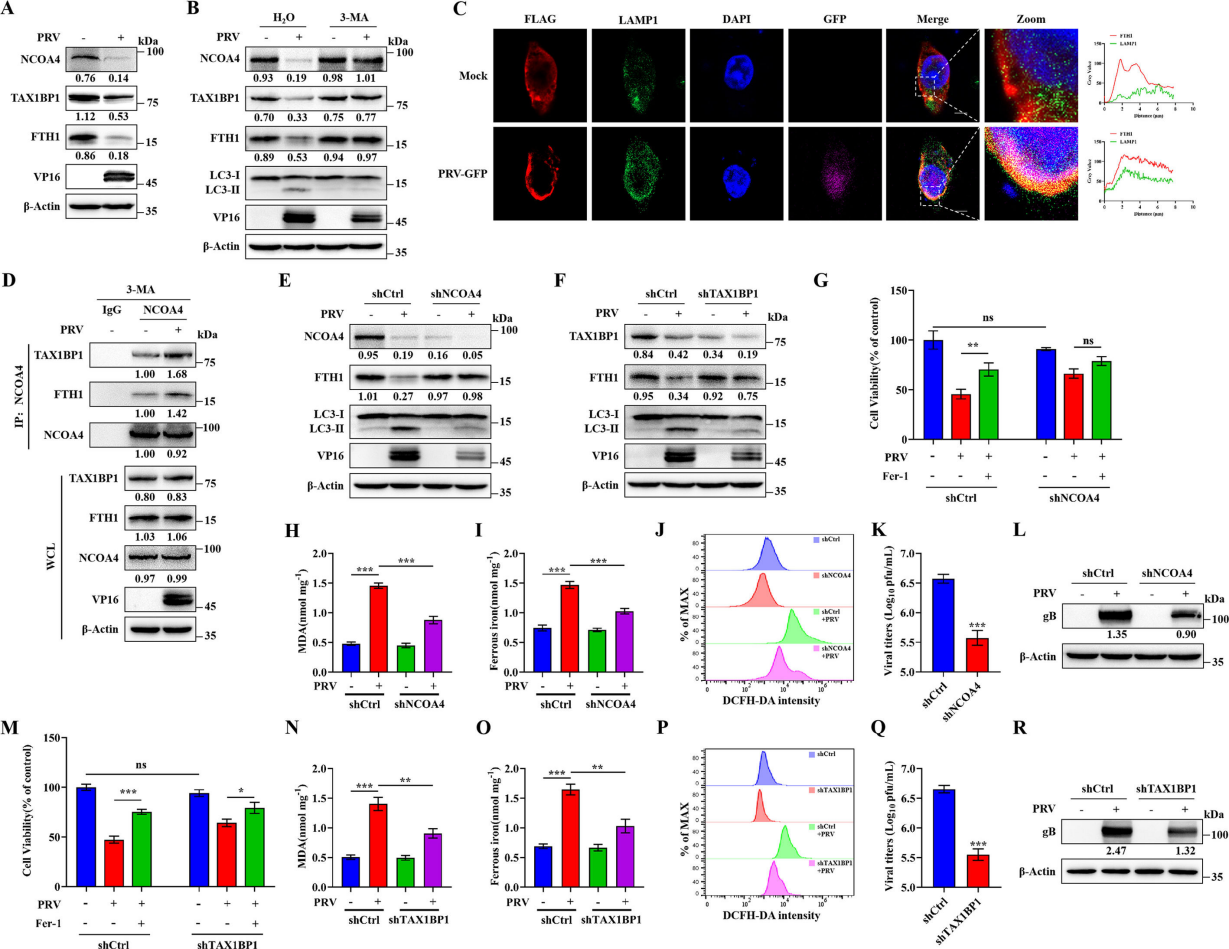
PRV induces ferritinophagy. (**A**) N2a cells were mock- or PRV-infected (MOI = 1) for 12 h. Cell lysates were analyzed by western blot for NCOA4, TAX1BP1, FTH1, VP16, and β-actin. (**B**) N2a cells were mock- or PRV-infected (MOI = 1) for 2 h and then treated with 5 mM 3-MA or the vehicle. At 12 hpi, cell lysates were analyzed by western blot for NCOA4, TAX1BP1, FTH1, LC3, VP16, and β-actin. (**C**) N2a cells were transfected with FLAG-FTH1 (1 µg) for 24 h, and then mock- or PRV-GFP-infected (MOI = 1). At 12 hpi, cells were stained for FTH1, LAMP1, and DAPI and observed by confocal microscopy. Scale bar, 10 µm. (**D**) N2a cells were mock- or PRV-infected (MOI = 1) for 2 h and then treated with 5 mM 3-MA or the vehicle. At 12 hpi, cell lysates were precipitated (co-IP) with anti-NCOA4 antibody and analyzed by western blot analysis for TAX1BP1, FTH1, NCOA4, VP16, and β-actin. The grayscale intensity was set at 1.00 for the mock-infected group. (**E**) NCOA4-knockdown and wild-type N2a cells were mock- or PRV-infected (MOI = 1) for 12 h. Cell lysates were analyzed by western blot for NCOA4, FTH1, LC3, VP16, and β-actin. (**F**) TAX1BP1-knockdown and wild-type N2a cells were mock- or PRV-infected (MOI = 1) for 12 h. Cell lysates were analyzed by western blot for TAX1BP1, FTH1, LC3, VP16, and β-actin. NCOA4-knockdown, TAX1BP1-knockdown, and wild-type N2a cells were mock- or PRV-infected (MOI = 0.1) for 24 h. NCOA4-knockdown (**G**) and TAX1BP1-knockdown (**M**) cells and wild-type N2a cells were treated with Fer-1 (80 µM) or vehicle (DMSO) for 2 h, followed by PRV infection (MOI = 0.1) or mock infection and additional treatment of Fer-1 or vehicle. At 24 hpi, cell viability was determined by CCK-8 assay, with the level of cell viability in cells treated with vehicle defined as 100%. MDA concentrations in NCOA4-knockdown (**H**) and TAX1BP1-knockdown (**N**) cell lysates were determined using MDA assay. Ferrous iron concentrations in NCOA4-knockdown (**I**) and TAX1BP1-knockdown (**O**) cell lysates were determined using a ferrous iron colorimetric assay. ROS levels in NCOA4-knockdown (**J**) and TAX1BP1-knockdown (**P**) cells were determined using a ROS assay. Cells and supernatants were harvested, and total virus titers of NCOA4-knockdown (**K**) and TAX1BP1-knockdown (**Q**) cells were determined in Vero cells using a plaque assay. Cell lysates of NCOA4-knockdown (**L**) and TAX1BP1-knockdown (**R**) cells were analyzed by western blot for gB and β-actin. The protein levels were quantified by Image J and normalized to β-actin. The data shown are means ± SD. *, *P* < 0.05; **, *P* < 0.01; ***, *P* < 0.001. The experimental data are representative of results from three independent experiments.

Next, we investigated the mechanism underlying FTH1 degradation. Typically, FTH1 degradation occurs through ferritinophagy (9). Therefore, we sought to elucidate the relationship between PRV infection and ferritinophagy. Ferritinophagy is a selective autophagy process mediated by the cargo receptor NCOA4, which binds ferritin and directs it to the autolysosome, playing a critical role in maintaining iron homeostasis. Additionally, the selective autophagy receptor TAX1BP1 directly interacts with NCOA4, facilitating ferritin transport to lysosomes for degradation (48). We analyzed the expression levels of NCOA4 and TAX1BP1 by western blot and observed that PRV infection induced concomitant degradation of NCOA4, TAX1BP1, and FTH1 in N2a cells, suggesting a specific role for these proteins in PRV-induced ferritin degradation (Fig. 7A). To confirm whether this process is autophagy-dependent, we treated cells with the autophagy inhibitor 3-MA. Without 3-MA treatment, NCOA4, TAX1BP1, and FTH1 were degraded following PRV infection; however, 3-MA treatment inhibited their degradation (Fig. 7B). During ferritinophagy, FTH1 is translocated to lysosomes, which are marked by LAMP1. We observed increased co-localization of FTH1 with the lysosomal marker LAMP1 after PRV-GFP infection by using confocal microscopy (Fig. 7C). We further examined the interaction between NCOA4, TAX1BP1, and FTH1 by co-immunoprecipitation. Our results showed that PRV infection enhanced the interaction among these proteins, leading to FTH1 degradation via ferritinophagy (Fig. 7D).

To investigate the roles of NCOA4 and TAX1BP1, we knocked down these proteins in N2a cells, with knockdown efficiency confirmed by RT-qPCR and western blot (Fig. S7B and C). In knockdown cells, FTH1 degradation was significantly reduced compared to controls (Fig. 7E and F), demonstrating that PRV-induced ferritinophagy depends on NCOA4 and TAX1BP1. Next, we infected NCOA4- and TAX1BP1-knockdown cells and wild-type cells with PRV to assess ferroptosis and viral replication. Knockdown of either NCOA4 or TAX1BP1 resulted in decreased ferroptosis markers (Fig. 7G through J and M through P) and reduced PRV replication (Fig. 7K, L, Q and R). Together, these findings indicate that PRV infection promotes the interaction of TAX1BP1, NCOA4, and FTH1, activating ferritinophagy and thereby increasing intracellular ferrous iron levels through ferritin degradation.

## DISCUSSION

Ferroptosis is an iron-dependent form of cell death characterized by intracellular iron overload, increased ROS production, and lipid peroxidation (49). Recent evidence has increasingly highlighted a strong link between ferroptosis and viral infections. In our study, we examined markers of apoptosis, necroptosis, pyroptosis, and ferroptosis following PRV infection and found that ferroptosis exhibited the most pronounced changes. Given the novelty of ferroptosis and the gaps in understanding its role in PRV infection, we focused our investigation on this form of cell death. We demonstrated that PRV, a member of the alpha-herpesvirus subfamily, induces ferroptosis in various cell lines by disrupting iron homeostasis through activation of TfR1 and induction of ferritinophagy.

Iron metabolism plays a central role in ferroptosis and can be broadly categorized into three components: iron uptake, storage, and efflux. Extracellular iron primarily enters the cytoplasm via transferrin receptor 1 (TfR1), while ferritin binds and stores iron, thereby regulating cytoplasmic ferrous iron levels. In this study, we demonstrated that PRV infection upregulates TfR1 expression by enhancing HIF-1β expression and promoting Rab11a-dependent endosomal trafficking of TfR1 to the cell membrane. This, in turn, increases iron uptake and elevates intracellular ferrous iron levels. Notably, TfR1 has been identified as an entry factor for several viruses, including rabies virus, hepatitis C virus (HCV), influenza A virus (IAV), and SARS-CoV-2 (50–53). Although our study focused on the role of TfR1 in iron uptake, its potential function as an entry receptor for alpha-herpesviruses warrants further investigation. Interestingly, in cells with knockdown of key ferroptosis-related genes, intracellular iron homeostasis remained relatively stable under physiological conditions. However, PRV infection caused substantial fluctuations in intracellular Fe^2+^ levels. Preliminary experiments revealed that treatment with the ferroptosis inhibitor Fer-1 suppressed PRV replication, suggesting that PRV may exploit ferroptosis to facilitate its replication. Moreover, manipulation of extracellular Fe³^+^ levels using DFOM and FAC demonstrated a positive correlation between iron concentration and PRV replication, providing additional insight into the virus’s reliance on iron metabolism.

Iron storage is closely linked to ferritinophagy, a process by which ferritin is degraded to release iron, especially under pathological conditions or when intracellular Fe^2+^ levels are low. In this study, we demonstrated that PRV infection induces ferritinophagy mediated by the selective autophagy receptors NCOA4 and TAX1BP1. NCOA4-driven ferritinophagy is essential for maintaining intracellular and systemic iron homeostasis and regulating iron-dependent physiological processes. Modulating NCOA4-mediated ferritinophagy can influence a cell’s sensitivity to iron-induced cell death. TAX1BP1, another selective autophagy receptor, plays a key role in host defense and innate immune regulation (54). It was initially identified as a binding partner of the HTLV-1 Tax protein (55) and has since been shown to interact with several viral proteins, including the measles virus N protein (56), papillomavirus E2 protein (57), and SARS-CoV-2 ORF3a (58). Our findings suggest that PRV exploits both NCOA4 and TAX1BP1 to activate ferritinophagy. Mechanistically, PRV facilitates the formation of complexes among TAX1BP1, NCOA4, and FTH1, promoting their transport to lysosomes. This process leads to ferritin degradation and the subsequent release of ferrous iron into the cytoplasm. Ferritinophagy is a complex biological process, and further research is needed to clarify how PRV proteins interact with NCOA4 and TAX1BP1. Additionally, it remains unclear why PRV benefits from inducing ferroptosis, especially the extensive plasma membrane damage observed during early infection. We hypothesize that PRV has evolved a mechanism to balance host cell death and rapid viral dissemination. By inducing ferroptosis, PRV may facilitate the early release of viral particles into the surrounding environment, allowing viral proteins to function more efficiently and enhancing both viral spread and immune evasion. This strategy could be particularly advantageous in the PRV lifecycle, where swift dissemination is critical for successful infection.

**Fig 8:**
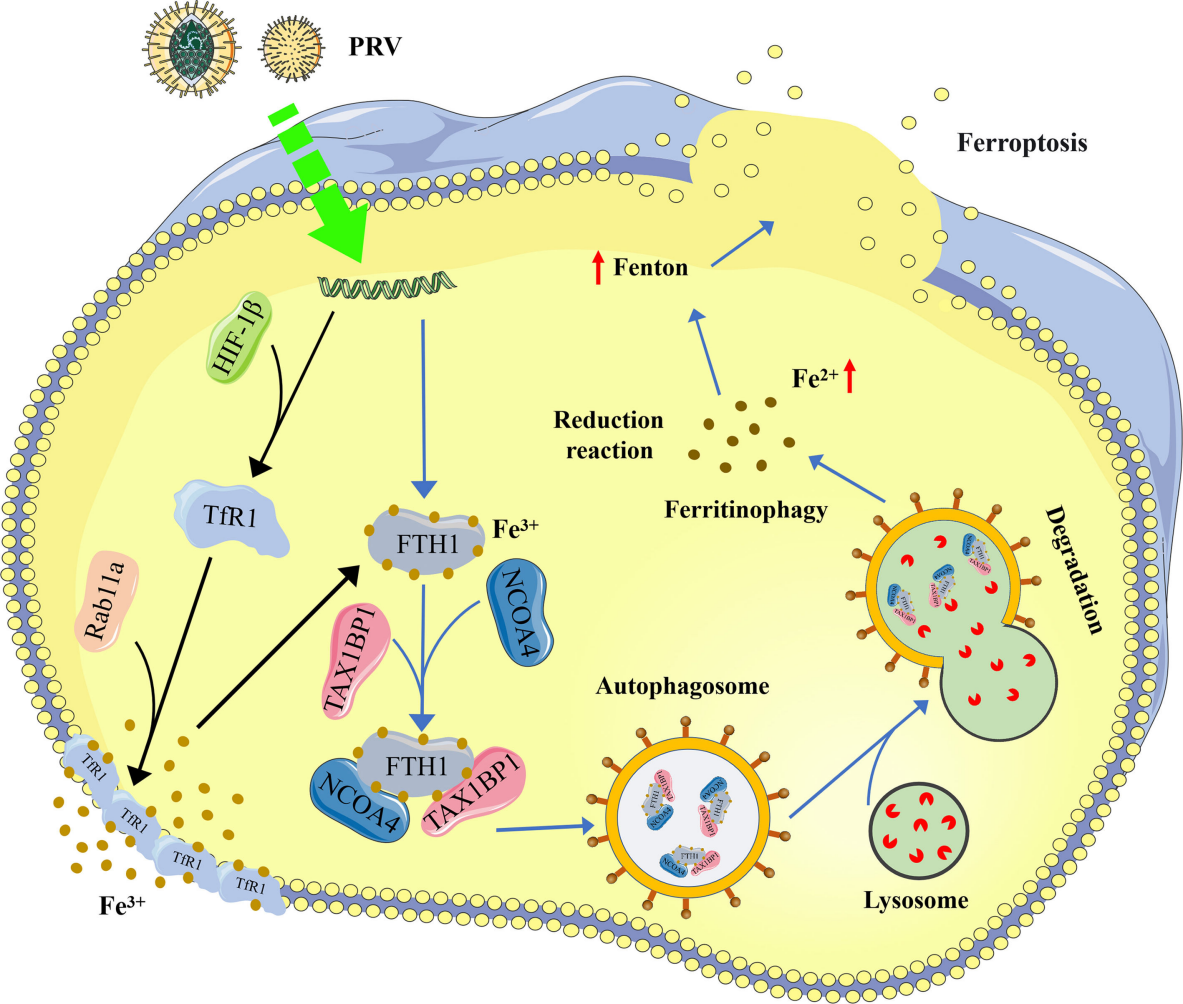
Schematic of the signaling pathway through which PRV infection disrupts cellular iron homeostasis, ultimately leading to the occurrence of ferroptosis. Black arrows indicate that PRV infection promotes the translocation of TfR1 to the cell membrane, increasing the influx of extracellular Fe^3+^. Blue arrows indicate that PRV infection utilizes the selective autophagy receptors NCOA4 and TAX1BP1 to degrade FTH1, inducing the process of ferritinophagy. This model was created by the author, with parts of the figure derived from Servier Medical Art (https://smart.servier.com), licensed under CC BY 4.0.

In conclusion, the proposed model of PRV replication facilitated by ferroptosis is illustrated in Fig. 8. Our findings provide new insights into the mechanism by which PRV induces ferroptosis and highlight its critical role in PRV pathogenesis. Moreover, these results suggest that targeting the ferroptosis pathway may represent a promising therapeutic strategy to inhibit herpesvirus replication.

## MATERIALS AND METHODS

### Cells and viruses

Human embryonic kidney-293T (HEK-293T, CRL-11268), Porcine kidney cell 15 (PK-15; CCL-33), Vero (CCL-81), SH-SY5Y (CRL-2266), and Neuro-2a (N2a; CCL-131) cells were obtained from the American Type Culture Collection (ATCC). PRV ZJ01 (GenBank: KM061380.1) is a prototype PRV strain used in this study as wild-type PRV (59). The recombinant virus PRV-GFP was constructed by inserting a GFP under the control of cytomegalovirus promoter into the genome of PRV ZJ01 by using the homogenous recombination. Preparation of viral stock and titration of infectivity were prepared using Vero cells.

### Antibodies and chemicals

The following primary antibodies were used: mouse anti-PRV-gB protein (produced in our laboratory), mouse anti-PRV-VP16 protein (produced in our laboratory), mouse anti-β-actin (Sigma-Aldrich, USA; no. A5441), mouse anti-TfR1 (Cell Signaling Technology, USA; no. 46222), mouse anti-TfR1 (Sigma-Aldrich, USA; no. MABC1765), rabbit anti-FTH1 (Cell Signaling Technology, USA; no. 3998), rabbit anti-IRP1 (Cell Signaling Technology, USA; no. 20272), rabbit anti-IRP2 (Cell Signaling Technology, USA; no. 37135), rabbit anti-HIF-1α (Cell Signaling Technology, USA; no. 14179), rabbit anti-HIF-1β (Cell Signaling Technology, USA; no. 5537), rabbit anti-Rab11a (Proteintech, USA; no. 20229-1-AP), mouse anti-NCOA4 (Santa Cruz Biotechnology, USA; no. sc-373739), rabbit anti-LAMP1 (Cell Signaling Technology, USA; no. 9091), rabbit anti-TAX1BP1 (Proteintech, USA; no. 14424-1-AP), rabbit anti-GSDME (Abcam, USA; no. ab215191), rabbit anti-GSDMD (Novus Biologicals, USA; no. NBP2-33422), rabbit anti-MLKL (Cell Signaling Technology, USA; no. 37705), and rabbit anti-p-MLKL (Affinity Biosciences, China; no. AF7420). Horseradish peroxidase (HRP)-labeled rabbit (Cat No. A0208) or mouse (Cat No. A0216) secondary antibodies were purchased from Beyotime (China). The following chemicals were used: RSL3 (Selleck, USA; no. S8155), Fer-1 (Selleck, USA; no. S7243), FAC (Selleck, USA; no. E0375), DFOM (Selleck, USA; no. S5742).

### Plasmid construction, transfection, and gene knockdown experiments

The FTH1 gene (NCBI Reference Sequence: NM_010239.2) was amplified from N2a cells (Mus musculus) by RT-PCR, the primers sequences are listed in Table S1. The amplified FTH1 fragment was digested by BamHI (Thermo Fisher, USA) and EcoRI (Thermo Fisher, USA) and then cloned into pCMV-N-FLAG (4,321, Beyotime, no. D2722), resulting in pCMV-FLAG-FTH1. The pLKO.1-TRC and lentivirus package plasmids pVSV-G (8,454, deposited by Bob Weinberg) and psPAX2 (12,260, deposited by Didier Trono) were purchased from Addgene. The shRNA for shTfR1, shFTH1, shHIF-1α, shHIF-1β, shIRP1, shIRP2, shRab11a, shNCOA4, and shTAX1BP1 was cloned into pLKO.1-TRC, resulting in pLKO.1-shTfR1, pLKO.1-shFTH1, pLKO.1-shHIF-1α, pLKO.1-shHIF-1β, pLKO.1-shIRP1, pLKO.1-shIRP2, pLKO.1-shRab11a, pLKO.1-shNCOA4, and pLKO.1-shTAX1BP1, respectively. The recombinant plasmid sequences were confirmed by DNA Sanger sequencing and then transfected into N2a cells or HEK-293T cells using Lipofectamine 3000 (Thermo Fisher, USA) according to the manufacturer’s instructions. For gene knockdown experiments, pLKO.1-shTfR1, pLKO.1-shFTH1, pLKO.1-shHIF-1α, pLKO.1-shHIF-1β, pLKO.1-shIRP1, pLKO.1-shIRP2, pLKO.1-shRab11a, pLKO.1-shNCOA4, and pLKO.1-shTAX1BP1 were co-transfected with lentivirus package plasmids (pVSV-G and psPAX2) into HEK-293T cells to generate lentiviruses. Subsequently, these lentiviruses were isolated and used to infect N2a cells. At 36 hpi (hours post infection), infected cells were cultured with 5 µg/mL puromycin to select knockdown cells. The efficiency of knockdown was assessed by RT-qPCR and western blot. The shRNA sequences are listed in Table S1.

### Transmission electron microscopy

N2a, PK-15, and SH-SY5Y cells were harvested and fixed with 2.5% glutaraldehyde at 4°C for 12 h. Next, they were treated with 1% osmium tetroxide, dehydrated in graded ethanol, and embedded in epoxy resin. Ultrathin sections were stained with uranyl acetate and lead citrate. Finally, sections were observed using a Hitachi TEM HT7700 (Hitachi, Tokyo, Japan). The diameters of mitochondria were measured by using ImageJ 1.8.0 software (National Institutes of Health).

### Cell viability assay

The levels of cell viability were determined using cell counting kit 8 (CCK-8) (Beyotime, China, no. C0038) according to the manufacturer’s instructions. Briefly, N2a, PK-15, and SH-SY5Y cells were seeded into 96-well plates and treated with different chemicals (RSL3, Fer-1, FAC, and DFOM) or PRV for 24 h. Then, 10 µL CCK-8 solution was added to each well. After incubating for 1 h, absorbance was measured at a wavelength of 450 nm by using microplate spectrophotometer (Agilent BioTek, USA).

### Cytotoxicity assay

The levels of cytotoxicity were determined using LDH Cytotoxicity Assay kit (Beyotime, China, no. C0016) according to the manufacturer’s instructions. Briefly, N2a, PK-15, and SH-SY5Y cells were seeded into 96-well plates and treated with RSL3 or PRV for 24 h. Then, 120 µL supernatant of each well was transferred into new 96-well plates. Sixty microliters of LDH working solution was added to each well. After incubating for 30 min, absorbance of each well was measured at a wavelength of 490 nm by using microplate spectrophotometer (Agilent BioTek, USA).

### Lipid peroxidation detection

Malondialdehyde (MDA) is an end-product of lipid peroxidation. The levels of MDA were determined using the Lipid Peroxidation MDA Assay Kit (Beyotime, China, S0131S) according to the manufacturer’s instructions. Briefly, N2a, PK-15, and SH-SY5Y cells were seeded into 6-well plates and treated with different chemicals (RSL3, FAC, and DFOM) or PRV for 24 h. Next, cells were lysed, and 100 µL of cell lysates from each well was mixed with 200 µL TBA working solution. After incubating for 15 min at 100°C, absorbance was measured at a wavelength of 532 nm by using microplate spectrophotometer (Agilent BioTek, USA).

### Lipid peroxidation analysis

The level of lipid peroxidation was determined using C11-BODIPY 581/591 (Thermo Fisher, USA, D3861) according to the manufacturer’s instructions. C11-BODIPY 581/591 was diluted to 10 mM with DMSO as a stock solution. N2a cells were seeded into a 6-well plate and treated with different titers of PRV for 24 h. Next, the C11-BODIPY 581/591 stock solution was diluted with DMEM at a 1:1,000 ratio to achieve a final concentration of 10 µM. The cell culture medium was then removed, and 1 mL of the diluted C11-BODIPY 581/591 solution was added to each well of the 6-well plate. Cells were incubated at 37°C for 30 min, followed by three washes with DMEM. Finally, cells were collected and analyzed by flow cytometry (Beckman Coulter, USA).

### Ferrous iron measurement

The levels of ferrous iron were determined using an iron assay kit (Sigma-Aldrich, USA, no. MAK025) according to the manufacturer’s instructions. Briefly, N2a, PK-15, and SH-SY5Y cells were seeded into 6-well plates and treated with different chemicals (RSL3, FAC, and DFOM) or PRV for 24 h. Then, the cells were lysed, and 80 µL cell lysates of each well was mixed with 80 µL working solution. After incubating for 30 min, absorbance of each well was measured at a wavelength of 490 nm by using microplate spectrophotometer (Agilent BioTek, USA).

### Reactive oxygen species detection

The levels of reactive oxygen species were determined using ROS Assay Kit (Beyotime, China, no. S0033) according to the manufacturer’s instructions. Briefly, N2a, PK-15, and SH-SY5Y cells were seeded into 6-well plates and treated with different chemicals (RSL3, FAC, and DFOM) or PRV for 24 h. Next, DCFH-DA has been diluted with DMEM at 1:1,000 to give a final concentration of 10 µM. Removed the cell culture medium and added 1 mL of diluted DCFH-DA for one well of a six-well plate. The cells were incubated in a cell incubator at 37°C for 20 min and then washed three times with DMEM. Cells were collected and analyzed by flow cytometry (Beckman Coulter, USA).

### Western blot

Total cell proteins were extracted using radioimmunoprecipitation assay (RIPA) buffer (20 mM Tris, pH 7.5, 150 mM NaCl, 1% Triton X-100, sodium pyrophosphate, β-glycerophosphate, EDTA, Na3VO4, and leupeptin). Protein concentrations were determined using bicinchoninic acid (BCA) assay according to the manufacturer’s instructions (Beyotime, China). After protein boiling and denaturation, an aliquot of proteins was separated by SDS-PAGE and transferred to polyvinylidene difluoride (PVDF) film (EMD Millipore, USA). The membrane was blocked for 2 h at room temperature with 5% skim milk in TBS-T (50 mM Tris-HCl, pH 7.6, 150 mM NaCl, and 0.1% Tween 20). Primary antibodies were added to the membrane, which was then incubated at 4°C overnight. The membrane was washed three times for 10 min with TBS-T and incubated with HRP-labeled anti-rabbit or anti-mouse antibodies for 1 h at room temperature. The membrane was washed three times for 10 min with TBS-T, and the bands were visualized using enhanced chemiluminescence (ECL) reagent and a gel imaging system (ImageQuant LAS 500; Cytiva). Finally, bands were quantitated using ImageJ 1.8.0 software (National Institutes of Health).

### Immunoprecipitation assay

Rabbit anti-TfR1, mouse anti-NCOA4, or normal IgG were mixed with Protein A + G Magnetic Beads (Beyotime, China, P2108) and incubated at room temperature for 1 h. Beads were washed three times with PBS (155.2 mM NaCl, 1.059 mM KH2PO4, 2.967 mM Na2HPO4). Cells were harvested and lysed with NP-40 buffer (50 mM Tris-HCl, pH 7.4, 1% NP-40, 150 mM NaCl, 1 mM EDTA, 1:400 protease inhibitor mixture). Supernatants were collected and mixed with pretreatment magnetic beads and incubated at 4°C for 6 h. Beads were washed three times with washing buffer (50 mM Tris-HCl, pH 7.4, 150 mM NaCl, 5 mM EDTA, 0.1% Triton X-100, and protease inhibitor mixture) and collected. The precipitated proteins were analyzed by western blot using the indicated antibodies.

### Flow cytometry

The FITC Annexin V Apoptosis Detection Kit I (BD Pharmingen, USA; no. 556547) was used to detect early stages of cell death. Briefly, cells were washed twice with PBS and resuspended in 1× binding buffer at a concentration of 1 × 10⁶ cells/mL. To 100 µL of the mixture (~10⁵ cells), 5 µL of FITC Annexin V and 5 µL of propidium iodide (50 µg/mL stock) were added, and the cells were incubated for 15 min at room temperature in the dark. After incubation, 400 µL of 1× binding buffer was added to each tube, and the cells were analyzed by flow cytometry. Three controls were included for flow cytometry analysis: (i) unstained cells, (ii) cells with FITC Annexin V but without PI, and (iii) cells without FITC Annexin V but with PI.

### Proximity ligation assay

Cells were either mock- or PRV-infected (MOI = 1) at various time points. PLA was performed using the Duolink *In Situ* Detection Reagents Green (Sigma-Aldrich, USA, no. DUO92014). Following the manufacturer’s instructions, cells were first subjected to fixation, recovery, and permeabilization. After blocking with Duolink blocking buffer at 37°C for 1 h, cells were incubated with mouse anti-TfR1 antibody and rabbit anti-Rab11a antibody at 37°C for 2 h. The cells were then treated with pre-diluted anti-rabbit and anti-mouse minus probes at 37°C for 1 h. Subsequently, the cells were incubated consecutively with 1× ligase and 1× polymerase for 30 and 100 min, respectively, and finally mounted on slides with Duolink *In Situ* Mounting Medium containing DAPI.

### RT-qPCR

Cells were harvested, and total RNA was extracted from PRV-infected or mock-infect cells using the Total RNA Kit I (Omega Bio-tek, USA, no. R6834). RNA was then reverse transcribed using a HiScript II 1st Strand cDNA Synthesis Kit (Vazyme, China, no. R211) according to the manufacturer’s instructions. RT-qPCR was performed with the AceQ qPCR SYBR Green Master Mix (Vazyme, China, no. Q112). The RT-qPCR primer sequences are listed in Table S1.

### Confocal experiments

Cells were fixed with 4% paraformaldehyde in PBS for 30 min at 4°C. After three washes with ice-cold PBS, cells were permeabilized with 0.1% Triton X-100 for 15 min and then blocked in 5% bovine serum albumin (BSA) in PBS for 1 h at 37°C. Cells were incubated with indicated primary antibodies (rabbit anti-TfR1, rabbit anti-LAMP1, or mouse anti-Flag) for 1 h at 37°C. Cells were washed with PBS and incubated with Alexa Fluor 594-conjugated goat anti-rabbit IgG (H + L) or Alexa Fluor 647-conjugated goat anti-mouse IgG (H + L) (Thermo Fisher, USA, no. A-11012/A-21235) for 1 h at 37°C in the dark and then stained with DAPI.

### Data analysis

All data were analyzed using GraphPad Prism 7.0 software (GraphPad Software, Inc.) and are presented as mean ± SD. Western blot band intensities were quantified using ImageJ 1.8.0 software (National Institutes of Health) and normalized to β-actin. Statistical comparisons were made using *t*-tests. Unless otherwise noted in the legends, *P*-values were calculated from three biological replicates. Data were reproduced in independent experiments as described in the legends.

## Data Availability

The data that support the findings of this study are available from the corresponding author upon reasonable request.

## SUPPLEMENTAL MATERIAL

Supplemental material jvi.00974-25-s0001.docx Figures S1 to S7; Table S1.

ASM does not own the copyrights to Supplemental Material that may be linked to, or accessed through, an article. The authors have granted ASM a non-exclusive, world-wide license to publish the Supplemental Material files. Please contact the corresponding author directly for reuse.
