# Supplementary material for: Pseudorabies virus induces ferroptosis by disrupting iron homeostasis through activation of TfR1 and ferritinophagy
Source: J Virol. 2025 Sep 2;99(9):e00974-25. doi: 10.1128/jvi.00974-25 (PMC12456129; doi:10.1128/jvi.00974-25)
Supplement: Supplemental material — Figures S1 to S7; Table S1. [file jvi.00974-25-s0001.docx]

**Supplementary Figures and Legends**

**
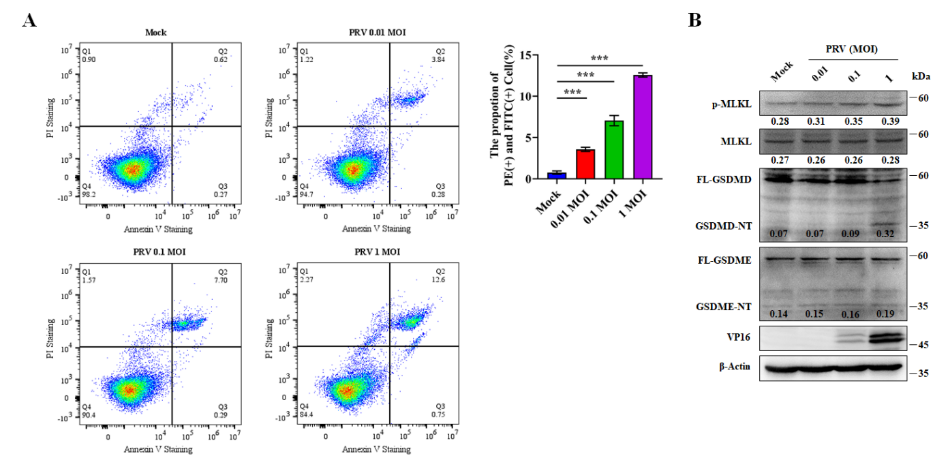
**

**Fig. S1 PRV induces distinct** **forms of programmed cell death.** **(A)** Apoptosis was assessed using an Annexin V-FITC/PI assay and analyzed by fluorescence-activated cell sorting (FACS) following mock or PRV infection (MOI = 0.01, 0.1, or 1) for 12 hours. The percentage of surviving cells is indicated in each panel. **(B)** MEF cells were mock- or PRV-infected (MOI = 0.01, 0.1, or 1) for 12 h. Cell lysates were subjected to western blot analysis for p-MLKL, MLKL, GSDMD, GSDME, VP16 and β-actin. Protein levels were quantified using Image J and normalized to β-actin. Data are presented as means ± SD. *, P < 0.05; **, P < 0.01; ***, P < 0.001. Representative results from three independent experiments are shown.


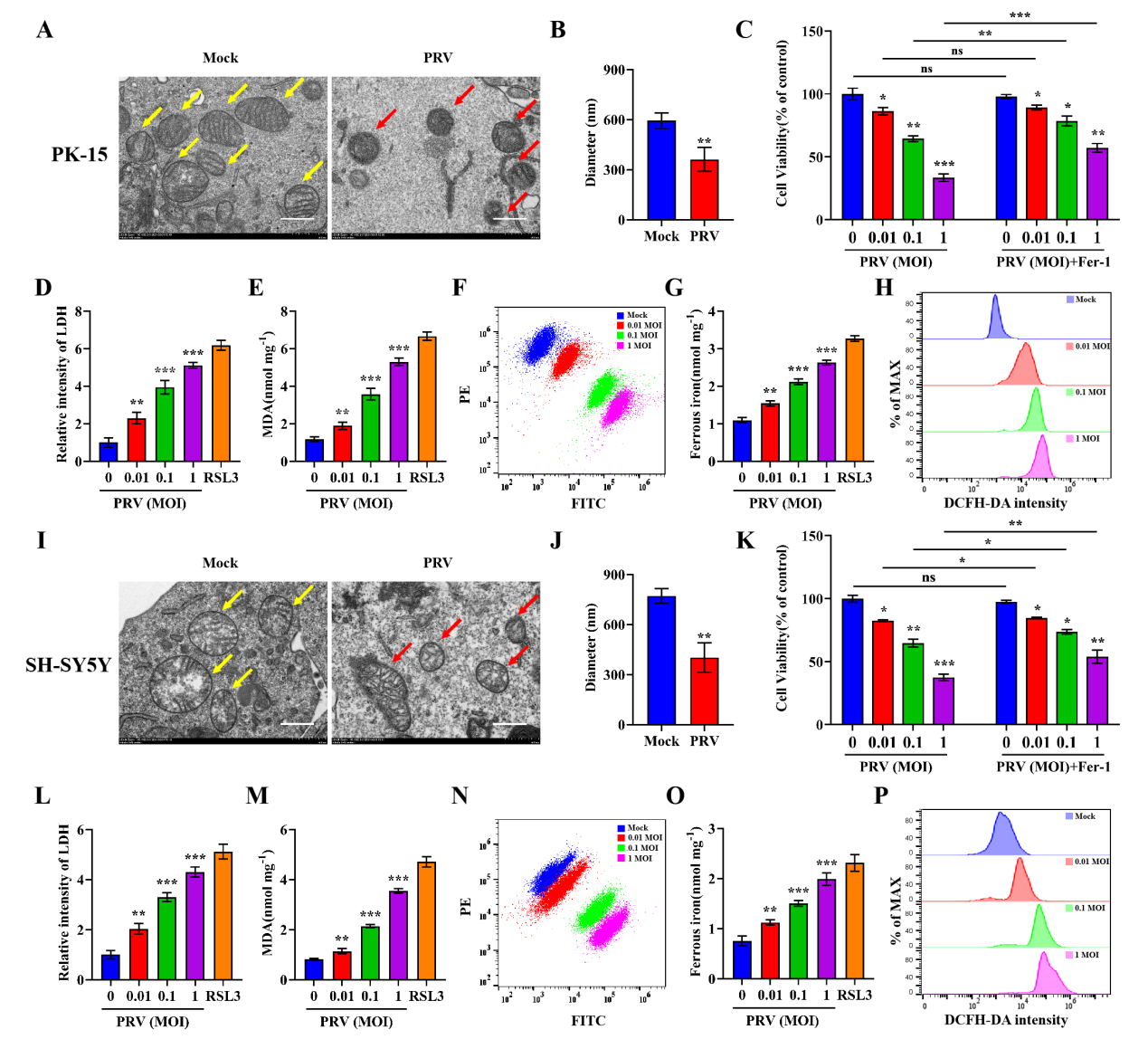


**Fig. S2 PRV induces ferroptosis in PK-15 and SH-SY5Y cells.** PK-15 **(A)** and SH-SY5Y **(I)** cells were mock- or PRV-infected (MOI = 0.1) for 24 h, and images were captured using a transmission electron microscope at 20,000 × magnification. Yellow arrows indicate normal mitochondria in mock-infected cells, while red arrows indicate mitochondrial atrophy in PRV-infected cells. Representative images are shown. Scale bar, 500 nm. Mitochondrial diameters in PK-15 (**B**) and SH-SY5Y (**J**) cells were quantified. PK-15 (**C**) and SH-SY5Y (**K**) cells were pretreated with ferrostatin-1 (Fer-1, 80 μM) or vehicle (DMSO) for 2 hours, followed by PRV infection (MOI = 0.1) or mock infection, and then maintained with Fer-1 or vehicle for an additional 24 hours. Cell viability was assessed using the CCK-8 assay, with vehicle-treated cells defined as 100%. PK-15 and SH-SY5Y cells were mock- or PRV-infected (MOI = 0.01, 0.1, or 1) for 24 hours, or treated with RSL3 (30 μM for PK-15; 20 μM for SH-SY5Y) as a positive control. Lactate dehydrogenase (LDH) release into the supernatants of PK-15 (**D**) and SH-SY5Y (**L**) cells was measured using a cytotoxicity assay, with LDH levels in mock-infected cells normalized to 1. Malondialdehyde (MDA) levels in PK-15 (**E**) and SH-SY5Y (**M**) cell lysates were determined using an MDA assay. Lipid peroxidation in PK-15 (**F**) and SH-SY5Y (**N**) cells was assessed using the fluorescent probe C11-BODIPY 581/591. Ferrous iron concentrations in PK-15 (**G**) and SH-SY5Y (**O**) cell lysates were measured using a colorimetric assay. Reactive oxygen species (ROS) levels in PK-15 (**H**) and SH-SY5Y (**P**) cells were measured using a ROS assay. Data are presented as means ± SD. *P < 0.05; **P < 0.01; ***P < 0.001. Results are representative of three independent experiments.


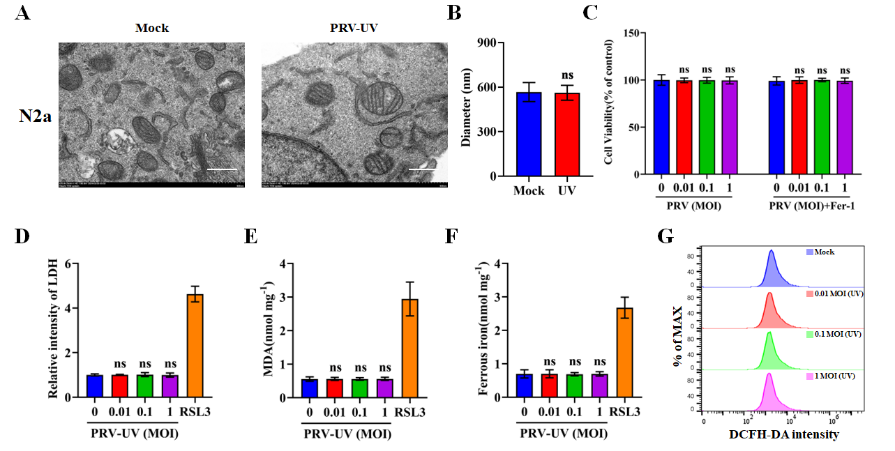
**Fig. S3 Inactivated PRV does not induce ferroptosis. (A)** N2a cells were mock-infected or infected with UV-inactivated PRV (PRV-UV, MOI = 0.1) for 24 hours. Images were captured using a transmission electron microscope at 20,000× magnification. Yellow arrows indicate normal mitochondria in mock-infected cells, while red arrows indicate mitochondrial atrophy in PRV-UV-infected cells. Representative images are shown. Scale bar, 500 nm. **(B)** Mitochondrial diameters in N2a cells were quantified. **(C)** N2a cells were pretreated with ferrostatin-1 (Fer-1, 80 μM) or vehicle (DMSO) for 2 hours, followed by PRV-UV or mock infection, and maintained with Fer-1 or vehicle for an additional 24 hours. Cell viability was assessed using the CCK-8 assay, with vehicle-treated cells defined as 100%. N2a cells were also mock- or PRV-UV-infected (MOI = 0.01, 0.1, or 1) for 24 hours, or treated with RSL3 (10 μM) as a positive control. **(D)** Lactate dehydrogenase (LDH) release into the supernatant was measured using a cytotoxicity assay, with LDH levels in mock-infected cells normalized to 1. **(E)** Malondialdehyde (MDA) levels in cell lysates were measured using an MDA assay. **(F)** Ferrous iron concentrations in cell lysates were determined using a colorimetric assay. **(G)** Intracellular reactive oxygen species (ROS) levels were assessed using a ROS assay. Data are presented as means ± SD. ns, not significant. Results are representative of three independent experiments.


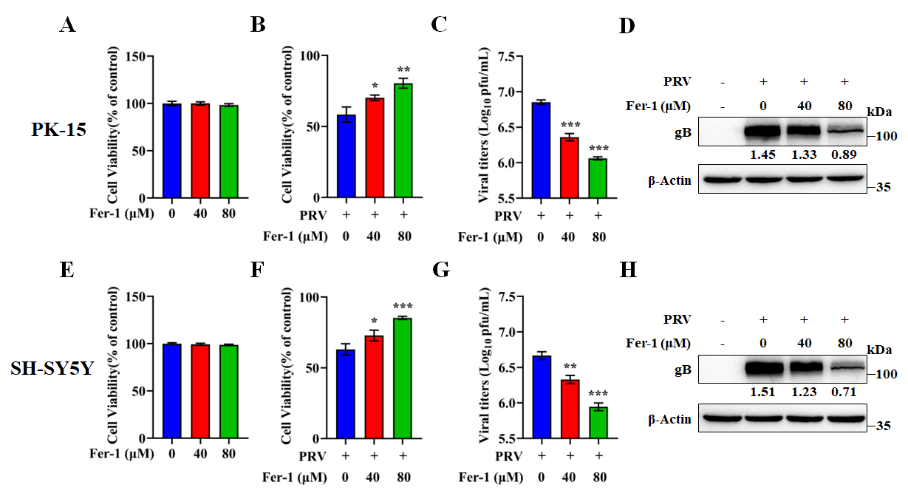


**Fig. S4 Ferroptosis promotes PRV replication in PK-15 and SH-SY5Y cells.** Cells were treated with different concentrations of ferrostatin-1 (Fer-1; 40 or 80 μM) or the vehicle (DMSO) for 24 h. (**A** and **E**) Cell viability of PK-15 (A) and SH-SY5Y (E) cells, respectively, was assessed using the CCK-8 assay, with viability in vehicle-treated cells defined as 100%. (**B** and **F**) PK-15 (B) and SH-SY5Y (F) cells were pretreated with Fer-1 (40 or 80 μM) or vehicle for 2 hours, followed by mock or PRV infection (MOI = 0.1) and continued treatment with Fer-1 or vehicle. At 24 hours post-infection (hpi), cell viability was measured using the CCK-8 assay. (**C** and **G**) At 24 hpi, cells and supernatants were collected, and viral titers in PK-15 (C) and SH-SY5Y (G) cells were determined in Vero cells using a plaque assay. (**D** and **H**) Cell lysates from PK-15 (D) and SH-SY5Y (H) cells were analyzed by western blot for PRV glycoprotein B (gB) and β-actin. Protein levels were quantified using ImageJ and normalized to β-actin. Data are presented as means ± SD. *P < 0.05; **P < 0.01; ***P < 0.001.


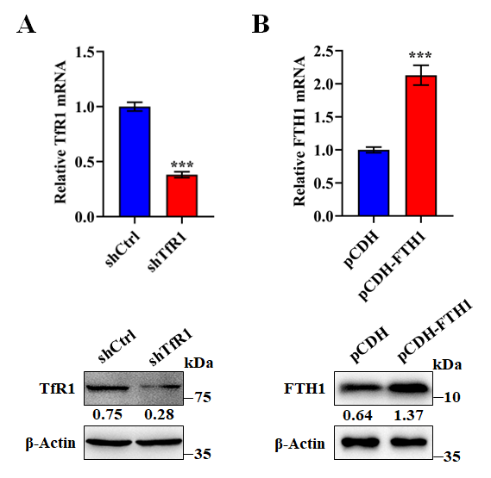


**Fig. S5 Efficiency of TfR1 knockdown and FTH1 overexpression.** (**A**) Wild-type and TfR1-knockdown N2a cells were harvested for RNA extraction and analyzed by RT-PCR for TfR1 and RNA18s rRNA expression. Protein levels of TfR1 and β-actin were assessed by western blot. (**B**) Wild-type and FTH1-overexpressing N2a cells were similarly harvested for RT-PCR analysis of FTH1 and RNA18s rRNA, and western blot analysis of FTH1 and β-actin. Protein levels were quantified using ImageJ and normalized to β-actin. Data are presented as mean ± SD. *P < 0.05; **P < 0.01; ***P < 0.001. Representative results from three independent experiments are shown.


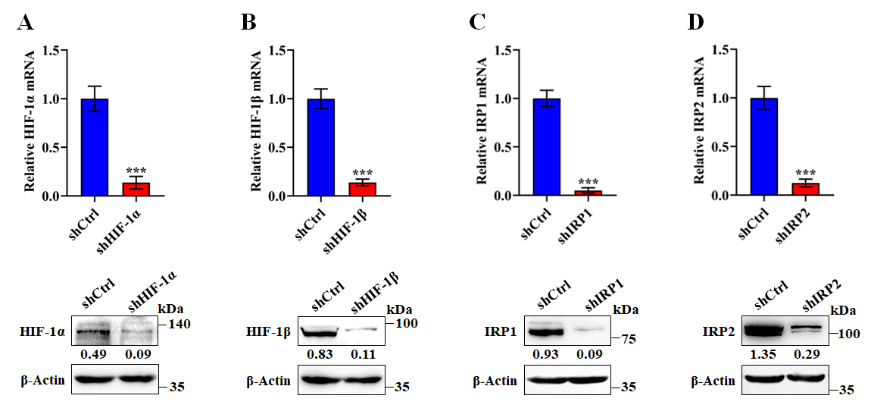


**Fig. S6 Knockdown efficiency of HIF-1α, HIF-1β, IRP1, and IRP2.** (**A-D)** Wild-type and knockdown N2a cells targeting HIF-1α (A), HIF-1β (B), IRP1 (C), and IRP2 (D) were collected for RNA extraction and subjected to RT-PCR analysis for the respective genes and RNA18s rRNA. Corresponding cell lysates were analyzed by western blot for HIF-1α (A), HIF-1β (B), IRP1 (C), or IRP2 (D), along with β-actin as a loading control. Data are presented as mean ± SD. *P < 0.05; **P < 0.01; ***P < 0.001. Results are representative of three independent experiments.

**
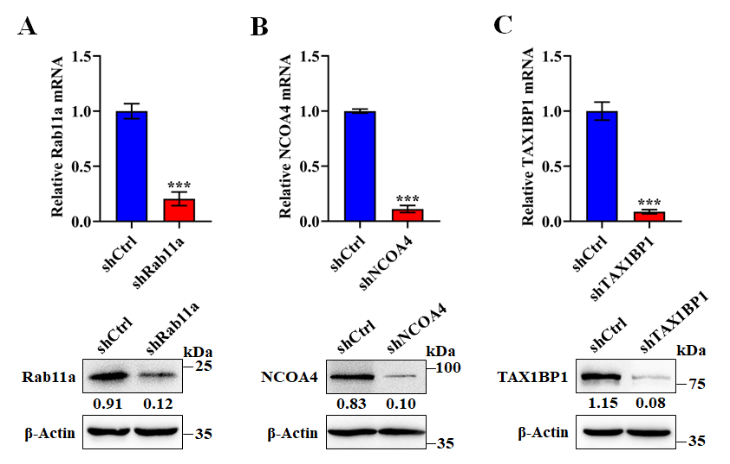
**

**Fig. S7 Knockdown efficiency of Rab11a, NCOA4 and TAX1BP1.** (**A-C**) Wild-type and knockdown N2a cells targeting Rab11a (A), NCOA4 (B), and TAX1BP1 (C) were collected for RNA extraction and subjected to RT-PCR analysis for the respective genes and RNA18s rRNA. Corresponding cell lysates were analyzed by western blot for Rab11a (A), NCOA4 (B), and TAX1BP1 (C), along with β-actin as a loading control. Data are presented as mean ± SD. *P < 0.05; **P < 0.01; ***P < 0.001. Results are representative of three independent experiments.

**Table S1. Primers used in this study**

| Genes | Primer Sequence 5’ to 3’ |
| --- | --- |
| Mouse FTH1-F | ATAGGATCCATGACCACCGCGTCTCCCTCGCAAG |
| Mouse FTH1-R | ATAGAATTCTTAGCTCTCATCACCGTGTCCCAGG |
| pLOK.1-shTfR1-F | CCGGGGAGACAGACAAGTCAGAAACCTCGAGGTTTCTGACTTGTCTGTCTCCTTTTTG |
| pLOK.1-shTfR1-R | AATTCAAAAAGGAGACAGACAAGTCAGAAACCTCGAGGTTTCTGACTTGTCTGTCTCC |
| pLOK.1-shFTH1-F | CCGGTGTATGCCTCCTACGTCTATCCTCGAGGATAGACGTAGGAGGCATACATTTTTG |
| pLOK.1-shFTH1-R | AATTCAAAAATGTATGCCTCCTACGTCTATCCTCGAGGATAGACGTAGGAGGCATACA |
| pLOK.1-shNCOA4-F | CCGGCGATCTCATCTATCAGCTTAACTCGAGTTAAGCTGATAGATGAGATCGTTTTTG |
| pLOK.1-shNCOA4-R | AATTCAAAAACGATCTCATCTATCAGCTTAACTCGAGTTAAGCTGATAGATGAGATCG |
| pLOK.1-shHIF-1α-F | CCGGTGGATAGCGATATGGTCAATGCTCGAGCATTGACCATATCGCTATCCATTTTTG |
| pLOK.1-shHIF-1α-R | AATTCAAAAATGGATAGCGATATGGTCAATGCTCGAGCATTGACCATATCGCTATCCA |
| pLOK.1-shHIF-1β-F | CCGGCCAAGACTCGTTCTTCCCAATCTCGAGATTGGGAAGAACGAGTCTTGGTTTTTG |
| pLOK.1-shHIF-1β-R | AATTCAAAAACCAAGACTCGTTCTTCCCAATCTCGAGATTGGGAAGAACGAGTCTTGG |
| pLOK.1-shIRP1-F | CCGGCGTCAGGATTGACTTCGAGAACTCGAGTTCTCGAAGTCAATCCTGACGTTTTTG |
| pLOK.1-shIRP1-R | AATTCAAAAACGTCAGGATTGACTTCGAGAACTCGAGTTCTCGAAGTCAATCCTGACG |
| pLOK.1-shIRP2-F | CCGGGTACGAAATTGTGATGGCTTTCTCGAGAAAGCCATCACAATTTCGTACTTTTTG |
| pLOK.1-shIRP2-R | AATTCAAAAAGTACGAAATTGTGATGGCTTTCTCGAGAAAGCCATCACAATTTCGTAC |
| pLOK.1-shRab11a-F | CCGGTAACCTCCTGTCTCGATTTACCTCGAGGTAAATCGAGACAGGAGGTTATTTTTG |
| pLOK.1-shRab11a-R | AATTCAAAAATAACCTCCTGTCTCGATTTACCTCGAGGTAAATCGAGACAGGAGGTTA |
| pLOK.1-shTAX1BP1-F | CCGGGCCTTCTTGAAGTATCACAAACTCGAGTTTGTGATACTTCAAGAAGGCTTTTTG |
| pLOK.1-shTAX1BP1-R | AATTCAAAAAGCCTTCTTGAAGTATCACAAACTCGAGTTTGTGATACTTCAAGAAGGC |
| RT-qPCR-18s-F | TAATGGAATAGGACCGCGGTTC |
| RT-qPCR-18s-R | TGGCAAATGCTTTCGCTCTG |
| RT-qPCR-TfR1-F | AGGTTGCAAATGCCCAAAGC |
| RT-qPCR-TfR1-R | GAGCATGTCCAAAGAGTGCAAG |
| RT-qPCR-FTH1-F | ACTGGAACTGCACAAACTGG |
| RT-qPCR-FTH1-R | TTGCGTAAGTTGGTCACGTG |
| RT-qPCR-NCOA4-F | ACTCTTCTGAGGTGCAGTGATG |
| RT-qPCR-NCOA4-R | ATTTGCTGTTCAGCCCGAAG |
| RT-qPCR-HIF-1α-F | TTTTGGCAGCGATGACACAG |
| RT-qPCR-HIF-1α-R | AAGTGGCTTTGGAGTTTCCG |
| RT-qPCR-HIF-1β-F | AATGGCTGTGGATGAGAACG |
| RT-qPCR-HIF-1β-R | TCACATTGGTGTTGGTGCAG |
| RT-qPCR-IRP1-F | ACAATGACCGCAAGACGTTC |
| RT-qPCR-IRP1-R | TGGATTGCTGGTGTTTGTGC |
| RT-qPCR-IRP2-F | TGTCAGTGGTCCAAAAAGGC |
| RT-qPCR-IRP2-R | TTCTGCTGCAACTTGGAAGC |
| RT-qPCR-Rab11a-F | ACCGCATTGTTTCTCAGAAGC |
| RT-qPCR-Rab11a-R | ACTGCACCTTTGGCTTGTTC |
| RT-qPCR-TAX1BP1-F | AAGGAGCAACTTCGCAAAGC |
| RT-qPCR-TAX1BP1-R | TCGCACATTCACTGCATCAC |
